# Mitophagy is induced in human engineered heart tissue after simulated ischemia and reperfusion

**DOI:** 10.1242/jcs.263408

**Published:** 2025-03-19

**Authors:** Mireia Nàger, Kenneth B. Larsen, Zambarlal Bhujabal, Trine B. Kalstad, Judith Rössinger, Truls Myrmel, Florian Weinberger, Asa B. Birgisdottir

**Affiliations:** ^1^ Division of Cardiothoracic and Respiratory Medicine, University Hospital of North Norway, 9019 Tromsø, Norway; ^2^ Department of Clinical Medicine, UiT-The Arctic University of Norway, 9019 Tromsø, Norway; ^3^ Department of Medical Biology, UiT-The Arctic University of Norway, 9019 Tromsø, Norway; ^4^ Department of Experimental Pharmacology and Toxicology, University Medical Center Hamburg Eppendorf, 20251 Hamburg, Germany; ^5^ DZHK (German Center for Cardiovascular Research), partner site Hamburg/Kiel/Lübeck, 20251 Hamburg, Germany; ^6^ Centro Nacional de Investigaciones Cardiovasculares Carlos III (CNIC), 28029 Madrid, Spain

**Keywords:** Engineered heart tissue, hiPSC, Ischemia–reperfusion, Mitochondria, Mitophagy

## Abstract

The paradoxical exacerbation of cellular injury and death during reperfusion remains a problem in the treatment of myocardial infarction. Mitochondrial dysfunction plays a key role in the pathogenesis of myocardial ischemia and reperfusion injury. Dysfunctional mitochondria can be removed by mitophagy, culminating in their degradation within acidic lysosomes. Mitophagy is pivotal in maintaining cardiac homeostasis and emerges as a potential therapeutic target. Here, we employed beating human engineered heart tissue (EHT) to assess mitochondrial dysfunction and mitophagy during ischemia and reperfusion simulation. Our data indicate adverse ultrastructural changes in mitochondrial morphology and impairment of mitochondrial respiration. Furthermore, our pH-sensitive mitophagy reporter EHTs, generated by a CRISPR/Cas9 endogenous knock-in strategy, revealed induced mitophagy flux in EHTs after ischemia and reperfusion simulation. The induced flux required the activity of the protein kinase ULK1, a member of the core autophagy machinery. Our results demonstrate the applicability of the reporter EHTs for mitophagy assessment in a clinically relevant setting. Deciphering mitophagy in the human heart will facilitate development of novel therapeutic strategies.

## Teaser

**Summary:** Mitochondrial dysfunction and lysosomal degradation of mitochondria (mitophagy) is induced after ischemia and reperfusion simulation in human engineered heart tissue, as shown with an endogenous pH-sensitive mitophagy reporter.

## INTRODUCTION

The heart is a highly energy demanding organ relying on ATP production to sustain its continuous contractions. The contractile cells of the heart (cardiomyocytes) produce vast amounts of ATP through oxidative phosphorylation (OXPHOS) within their mitochondria. Here, protein complexes of the electron transport chain in the inner mitochondrial membrane play a major role and oxygen acts as the terminal electron acceptor (Nolfi-Donegan et al., 2020). Hence, cardiomyocytes are highly dependent on mitochondria and oxygen for energy production. An imbalance between oxygen demand and supply results in myocardial ischemia and is caused by a reduction or blockage of blood flow to the heart. Prolonged ischemia leads to myocardial infarction and the size of the infarct is correlated to the duration of ischemia (Christian et al., 1992; Hasche et al., 1995; Thygesen et al., 2019). Notably, ischemic heart disease is the leading cause of death worldwide (Nowbar et al., 2019).

Ischemic heart tissue suffers reduced oxygen availability (hypoxia), limited metabolic substrate accessibility and inadequate removal of metabolic waste products; thus, cardiomyocyte function is affected. Cellular hallmarks of ischemia include an altered energy metabolism towards anaerobic glycolysis, and changes in mitochondria morphology and cell injury due to focal plasma membrane disruption, resulting in the release of enzymes and subcellular components (Jennings and Reimer, 1991). Restoration of blood flow after ischemia is termed reperfusion and timely reperfusion is the best therapeutic option (Byrne et al., 2023). However, reperfusion can concurrently further aggravate damage to ischemia-compromised cardiomyocytes leading to cardiomyocyte death. These conjugated events are termed ischemia/reperfusion injury and involve cellular processes such as Ca^2+^ and proton overload, oxidative stress and mitochondrial dysfunction (Nowbar et al., 2019).

Mitochondria play a key role in myocardial ischemia and reperfusion injury due to their central role in energy generation, signaling, ROS production and programmed cell death (Hernandez-Resendiz et al., 2023). Dysfunctional mitochondria are detrimental to cells and must be removed to prevent further damage. Selective autophagy of dysfunctional or superfluous mitochondria, termed mitophagy, involves the sequestration of mitochondria, or parts of mitochondria, into double-membraned vesicles (autophagosomes) and culminates in lysosomal degradation of their contents (Kim et al., 2007; Lemasters, 2005; Youle and Narendra, 2011). Numerous mitophagy pathways have been identified, but mechanistic insight into mitophagy in response to different cellular stress is still limited (Ganley and Simonsen, 2022). The unc-51 like autophagy activating kinase 1 (ULK1) protein kinase mediates autophagy initiation and has been implicated in mitophagy (Dhingra et al., 2019; Egan et al., 2011; Laker et al., 2017; Murakawa et al., 2019; Wu et al., 2014). The most-studied mitophagy pathway is PINK-PARKIN-dependent mitophagy, driven by the enzyme 3 (E3) ubiquitin ligase PARKIN or parkin RBR E3 ubiquitin protein ligase (PRKN) and the protein kinase PTEN-induced putative kinase 1 (PINK1) upon mitochondrial depolarization (Narendra and Youle, 2024). Recently, small-molecule activation of PARKIN and enhanced PARKIN-dependent mitophagy has been shown to mediate cardioprotection in mice (Ai et al., 2025). BCL2-interacting protein 3 (BNIP3) acts as a mitophagy receptor in the mitochondrial outer membrane (Hanna et al., 2012). BNIP3 is a transcriptional target of the transcription factor hypoxia inducible factor (HIF) 1α (HIF1α) and is induced during hypoxia to activate mitophagy without requiring mitochondrial depolarization (He et al., 2022; Zhao et al., 2020). Like PARKIN, BNIP3 has also been linked to mitophagy regulation in the heart (Hamacher-Brady et al., 2006; Marzook et al., 2024). Notably, both PARKIN and BNIP3 can be phosphorylated by ULK1 (Hung et al., 2021; Poole et al., 2021).

Mitophagy during ischemia and reperfusion is highly regulated, but the underlying mechanisms remain mostly elusive. Mitophagy is upregulated during ischemia in mice (Saito et al., 2019; Zhou et al., 2018). Conversely, reports indicate both induction (Lu et al., 2016) and inhibition (Zhang et al., 2021; Zhou et al., 2018) of mitophagy during reperfusion. In these studies, perturbation of mitophagy after ischemia and reperfusion is demonstrated through gene knockout studies of proposed regulators in mice. Impairment of mitophagy during reperfusion is considered to promote injury whereas induction of mitophagy confers cardioprotection. Furthermore, inhibiting the overactivation of mitophagy during reperfusion is regarded as beneficial (Xu et al., 2023).

Preclinical studies of the role of mitophagy in myocardial ischemia and reperfusion commonly involve conventional cell cultures (mostly murine) and small animals (mostly rodents) (Xu et al., 2023). Hence, deciphering the level, mechanistic details and regulation of mitophagy during ischemia and reperfusion in the human heart are, for a large part, unsolved tasks. Engineered heart tissue (EHT) generated with beating cardiomyocytes derived from human-induced pluripotent stem cells (hiPSCs) offers a unique opportunity to study human cardiac physiology and pathophysiology (Tenreiro et al., 2021; Weinberger et al., 2017). In EHTs, hiPSC-derived cardiomyocytes are embedded in a fibrin-based matrix between flexible silicone posts that deflect with each beat, allowing the EHT to contract auxotonically and perform contractile work. This leads to advanced morphological maturation of the cardiomyocytes resulting in a small, three-dimensional (3D) beating tissue kept in a standard cell culture dish (Breckwoldt et al., 2017).

In this paper, we simulated ischemia and reperfusion in EHTs and studied the effects on cell injury and mitochondrial morphology, as well as the impact on mitochondria function and their lysosomal degradation. For visualization and direct assessment of mitophagy, we employed CRISPR/Cas9 endogenous gene editing of hiPSCs, generating a cell line with a pH-sensitive mitophagy reporter. EHTs derived from this cell line enabled monitoring of the basal mitophagy level, a challenging task to conduct in tissue using traditional biochemical assays (Klionsky et al., 2021). Furthermore, we detected an induction of mitophagy flux in EHTs after ischemia and reperfusion simulation and demonstrated the importance of ULK1 activity. To our knowledge, this is the first report on alterations in mitophagy flux in a human 3D cardiac tissue model subjected to ischemia and reperfusion simulation.

## RESULTS

### Ischemia and ischemia/reperfusion simulation in EHTs lead to cell injury and cell death

**Fig. 1.**
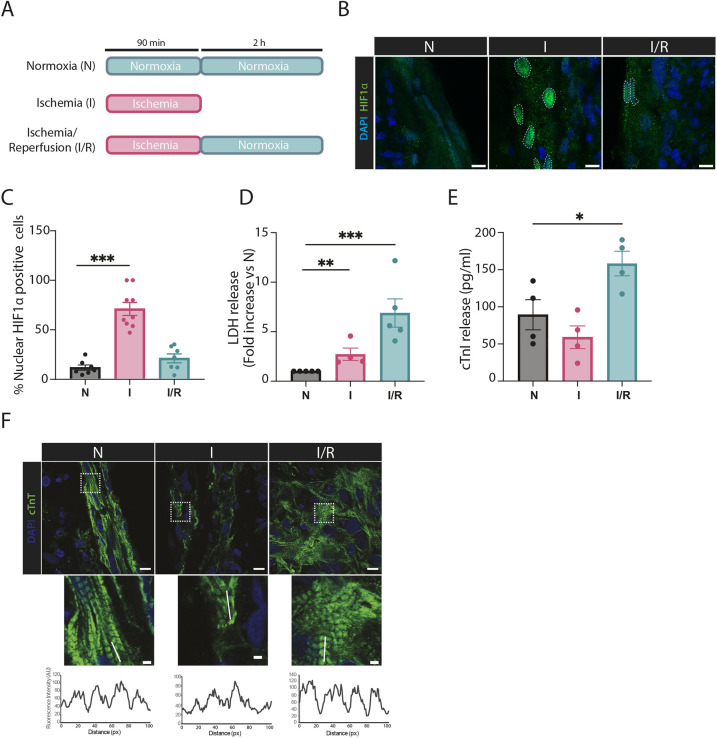
**I and I/R simulation induces HIF1α translocation and cell injury in EHTs.** (A) Study design displaying N, I and I/R experimental groups. EHTs were subjected to I for 90 min and I/R with R for 2 h after I. Blue corresponds to normal EHT culture medium in 40% O_2_. Red corresponds to lactate-based ischemia simulation medium (4 mM sodium lactate, 10 µg/ml insulin, 33 µg/ml aprotinin, 1% P/S and DMEM without glucose) in 0.3% O_2_. Time duration of the treatments is indicated. (B) EHTs were subjected to I and I/R and EHT sections were immunostained against HIF1α (green) and stained with DAPI (blue). Dashed areas indicate nuclear HIF1α. (C) The number of nuclear HIF1α-positive cells was quantified in all conditions. The data represents two independent experiments with at least eight ROIs analysed. (D) EHT medium was collected after N, simulation of I and I/R and analyzed for LDH content. Results are shown as a fold increase versus N in four independent experiments. (E) Release of cTnI in the medium after N, I and I/R. The data represents medium sample collections from four independent experiments. (F) Immunofluorescence staining against cTnT (green) was performed in EHT sections subjected to the N, I and I/R conditions. Sections were stained with DAPI (blue). Insets show magnified views of boxed areas indicated in the top images. A line profile in each inset demonstrates the periodicity or disarray of cTnT staining, representing sarcomere organization. The images are representative of three independent experiments. Data are represented as mean±s.e.m. *P<0.05, **P<0.01, ***P<0.005 (one-way ANOVA with Dunnett's test for multiple comparisons). For each independent experiment, the EHTs were generated from a distinct hiPSC differentiation. Scale bars: 10 µm (B; F, overview); 2 µm (F, inset).

EHTs, generated from a hiPSC line (UKEi003-C) from a donor without a diagnosed disease, were allowed to mature for at least 21 days to achieve coherent, synchronous beating. The EHTs were then subjected to simulated ischemia (I) by incubation for 90 min in culture medium without glucose, supplemented with lactate in hypoxic conditions (0.3% O_2_). To simulate reperfusion after ischemia (I/R), the EHTs were subsequently incubated for 2 h in normal growth medium in normoxic conditions. Time-matched control EHTs remained in normal growth conditions and normoxia (N) (Fig. 1A). To verify that the simulation treatments led to detectable cellular effects correlated with I, we first assessed the cellular localization of HIF1α in vibratome sections of fixed EHTs. HIF1α is a key transcription factor regulating cell metabolism in response to low levels of available oxygen (Jaakkola et al., 2001; Semenza et al., 1991; Zhao et al., 2023) and is commonly used as a marker of hypoxia. Immunostaining against HIF1α revealed nuclear translocation of HIF1α, indicating HIF1α stabilization and nuclear activity only during I simulation (Fig. 1B,C). Release of lactate dehydrogenase (LDH) from cardiomyocytes is a hallmark of cell injury associated with I and I/R (Firth et al., 1994; Funcke et al., 2020). Thus, we analyzed LDH content in EHT medium after I and I/R simulation and observed a marked increase in LDH levels compared to what was detected in EHT medium from controls (Fig. 1D). Elevated blood levels of cardiac troponin (cTn), of the cTnI and cTnT subtypes (also known as TNNI3 and TNNT2, respectively), indicate cardiomyocyte injury and cell death, and are used in the clinic as an indicator of myocardial infarction (Mishra et al., 2019; Reichlin et al., 2011; Thygesen et al., 2019). Both cTn isoforms constitute an important part of the A band of sarcomeres in cardiomyocytes and their structural disarray is described during I and I/R simulations (Chen and Vunjak-Novakovic, 2019). We observed an increased level of cTnI in culture medium from I/R EHTs (Fig. 1E). Furthermore, immunostaining against cTnT, for visualizing A bands of sarcomeres, revealed disassembly within sarcomeres after I, which was only partially restored after reperfusion (Fig. 1F). Notably, α-actinin immunostaining of fixed EHT sections, for visualizing the Z lines of sarcomeres, was unchanged in the different treatment groups, most likely due to short I and I/R simulation time compared to that used in previously published data on EHT I/R simulation (Chen and Vunjak-Novakovic, 2019) (Fig. S1A). In addition, we did not detect altered sarcomere ultrastructure in transmission electron microscopy (TEM) images of the tissue sections (Fig. S1B). In summary, these data demonstrate that EHTs subjected to simulated I and I/R can be used as a model to study I/R damage in the human heart.

### Mitochondrial function in EHTs is perturbed during I and I/R

Cardiac I/R injury causes mitochondrial impairment that ultimately can lead to cell death and myocardial dysfunction (Hou et al., 2018). Additionally, I/R decreases mitochondrial fusion and induces mitochondrial fragmentation which results in energy production decrease (Tian et al., 2017).

**Fig. 2.**
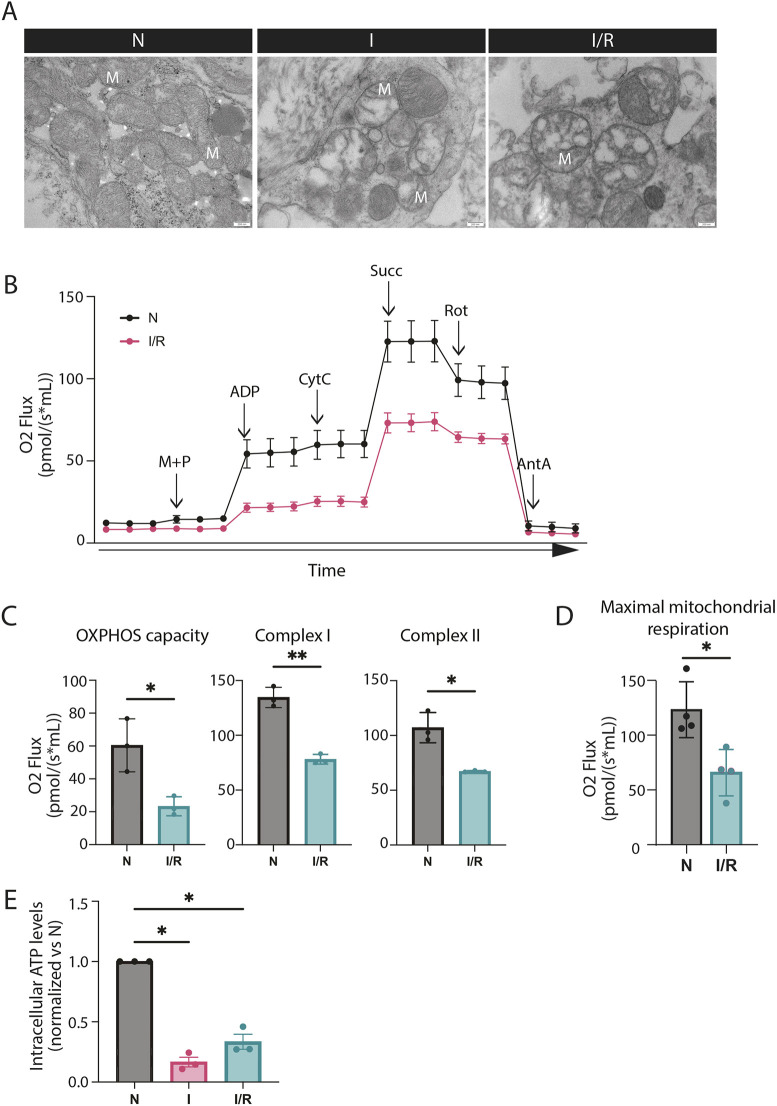
**I/R simulation induces mitochondrial damage and impaired energy production in EHTs.** (A) TEM images of EHTs subjected to N, I for 90 min and I/R with R for 2 h after I. The images are from a representative experiment of three independent experiments. M, mitochondria. Scale bars: 200 nm. (B) Representative oxygen consumption rates (OCR) in EHTs subjected to N and I/R with R for 2 h after I. Addition of 0.1 mM malate (M) and 5 mM pyruvate (P) indicates non-phosphorylating resting state (LEAK state). To test OXPHOS capacity 2.5 mM ADP was added. The integrity of the mitochondrial outer membrane was assessed by adding 10 µM cytochrome *c* (CytC); incorporation of 100 mM succinate (Succ) demonstrates complex I function; addition of 0.5 µM rotenone (Rot) indicates complex II functions. Treatment with 2.5 µM antimycin (AntA) indicates residual oxygen consumption. The data represents four independent experiments. (C) OCR values in EHTs subjected to N and I/R indicating OXPHOS capacity, complex I and complex II function. The data represents three independent experiments. (D) OCR values in EHTs subjected to N and I/R indicating maximal mitochondrial respiration. The data represent four independent experiments. (E) Intracellular ATP levels were measured in whole EHTs after N, I for 90 min and I/R with R for 2 h after I. Data are expressed as a fold increase relative to ATP values measured for EHTs in N. The data represents the average of two replicates by group in three independent experiments. Data are represented as mean±s.e.m. *P<0.05, **P<0.01 (one-way ANOVA with Dunnett's test for multiple comparisons). For each independent experiment the EHTs were generated with cardiomyocytes from a distinct hiPSC differentiation.

We investigated mitochondrial ultrastructure after I and I/R simulation by TEM of EHT sections. In control EHTs, we observed mitochondria with numerous tortuous cristae and homogenous electron-dense mitochondrial matrix, constituting typical characteristics of healthy mitochondria with high metabolic activity (Schaper and Schaper, 1983) (Fig. 2A). Intriguingly, mitochondria in I and I/R simulation groups, displayed swelling and reduced number of cristae. Furthermore, mitochondrial matrix clearing (electron-lucent areas within mitochondria) was particularly evident in the I/R group (Fig. 2A). Such ultrastructural alterations of mitochondria are widely accepted as hallmarks of I/R injury (Jennings and Ganote, 1974; Jennings and Reimer, 1991). In addition, immunostaining against the translocase of the outer mitochondrial membrane 20 (TOMM20) protein, in vibratome sections of fixed EHTs, indicated more disorganized and fragmented mitochondria after I and I/R, compared to what was seen for control EHTs (Fig. S1C).

To assess whether the detected detrimental morphological characteristics of the mitochondria correlated with impaired function, we explored mitochondrial respiration by applying high-resolution respirometry using an Oxygraph-2k (Oroborus Instruments). Here, we measured mitochondrial respiration in whole EHTs after I/R treatment and compared to the results of respiration measurements in control EHTs (N) (Fig. 2B). Assessment of mitochondrial respiration involves determining oxygen consumption, and thus the presence of oxygen in the medium is needed (Walsh et al., 2023). After I/R treatment, a significant decrease in OXPHOS capacity was detected (Fig. 2C). Additionally, there was a notable impairment in the functions of complex I and complex II, key components of the electron transport chain (Fig. 2C). Finally, maximal mitochondrial respiration capacity was significantly lower in the EHTs after I/R simulation compared to that measured in the control EHTs (N) (Fig. 2D). Considering these results, we assessed ATP production in the EHTs after I and I/R. As expected, we observed a decrease in ATP production after I simulation due to the limited amount of available oxygen for OXPHOS. Interestingly, after I/R simulation, mitochondrial ATP synthesis was only partially recovered as compared to the control group (N) (Fig. 2E). Taken together, these results demonstrate that I/R treatment of the EHTs results in damaged mitochondria with highly impaired ATP generation.

### Mitochondria proximity to lysosomes increases during I/R

**Fig. 3.**
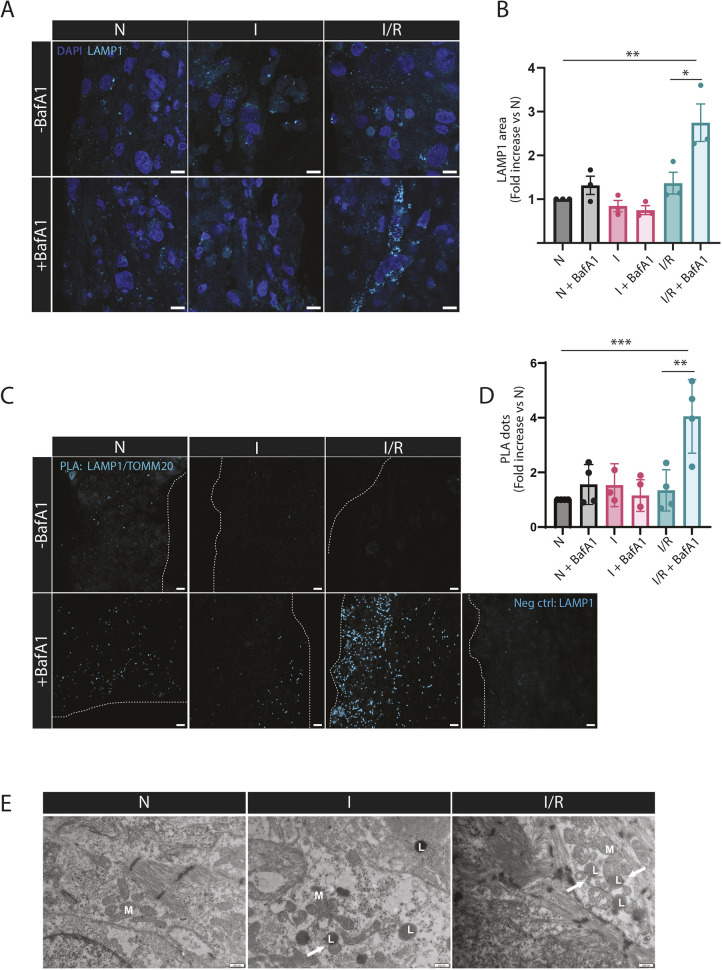
**I/R increases abundance of lysosomes and their proximity to mitochondria.** (A) EHTs were subjected to N, I for 90 min and I/R with R for 2 h after I and incubated with 200 nM BafA1 for 90 min during the I group, or the last 2 h in the N or I/R groups. Representative confocal images of EHT sections immunostained against LAMP1 (cyan) and stained with DAPI (blue). (B) The LAMP1-stained area in confocal images was quantified using ImageJ and Volocity. Results are displayed as fold increase versus the N group. The data represents three independent experiments. (C) Proximity of TOMM20 and LAMP1 was assessed using PLA in EHT sections of the different treatment groups. Each fluorescent signal (cyan) termed a PLA dot corresponds to where the TOMM20 protein is in <40 nm proximity to the LAMP1 protein. A negative control was performed with incubation of the LAMP1 antibody alone. Dashed lines indicate the EHT area. (D) The number of PLA dots was quantified in all conditions and represented as a fold increase versus the control group (N). The data represents three to four independent experiments. (E) Representative TEM images of EHTs subjected to N, I for 90 min and I/R. The data represent three independent experiments M, mitochondria; L, lysosomes. Arrows indicate mitochondria in proximity to lysosomes. Data are represented as mean±s.e.m. *P<0.05, **P<0.01, ***P<0.005 (one-way ANOVA with Dunnett's test for multiple comparisons). For each independent experiment the EHTs were generated from cardiomyocytes from a distinct hiPSC differentiation. Scale bars: 10 µm (A,C); 200 nm (E).

Dysfunctional mitochondria present a threat to cellular health and can be eliminated through lysosomal degradation (Twig et al., 2008; Youle and Narendra, 2011). An increase in the number of autophagosomes accompanied by an increase in lysosomes implicates either an induction or inhibition of autophagic flux. To distinguish this, the lysosomal inhibitor bafilomycin A1 (BafA1) can be applied. BafA1 targets the vacuolar H^+^-ATPase (V-ATPase), and thus elevates lysosomal pH and also inhibits fusion between autophagosomes and lysosomes. A further accumulation of autophagosomes or autolysosomes in the presence of BafA1 implies autophagy flux induction (Klionsky et al., 2021; Mauvezin and Neufeld, 2015). We analyzed the abundance of lysosomes in the EHTs after I and I/R simulation by immunostaining against lysosomal-associated membrane protein 1 (LAMP1). Interestingly, we observed a substantial increase in LAMP1 staining with BafA1 treatment during reperfusion compared to what was detected upon I/R without the inhibitor. However, the level of LAMP1 staining was not significantly altered in the control (N) or the I group in the presence of BafA1 (Fig. 3A,B). Notably, western blot analysis of the EHTs revealed an increase in the level of the lipidated form of microtubule associated protein 1 light chain β (MAP1LC3B) protein (LC3-II; a major autophagosome marker) after I/R in the presence BafA1, indicative of an accumulation of autophagosomes. Furthermore, the level of the autophagy receptor p62 (also known as SQSTM1) was decreased after I/R (Fig. S1D). Thus, these results indicate that the autophagy flux is induced after I/R simulation in the EHTs.

To further unravel lysosomal degradation of mitochondria during I/R in the EHTs, we assessed the proximity of mitochondria and lysosomes using a proximity ligation assay (PLA) (Fredriksson et al., 2002). To that end, we performed PLA with antibodies against TOMM20 (mitochondria) and LAMP1 (lysosomes) respectively (Fig. 3C) on fixed EHT sections. Statistical analysis revealed no significant differences in the number of PLA signals between the N, I and I/R groups. However, in the presence of BafA1, we observed a more than twofold increase of PLA signals in the I/R group (I/R versus I/R+BafA1) (Fig. 3D). In contrast, we detected only a small increase in the N group (N versus N+BafA1) and no difference in the I group (I versus I+BafA1) (Fig. 3D). Interestingly, TEM images of the EHT sections also indicated a more pronounced proximity of mitochondria and lysosomes after I/R (Fig. 3E). In summary, these findings suggest that lysosomal degradation of mitochondria is induced during reperfusion, but not during ischemia.

### Cardiomyocytes with a pH-sensitive mitochondrial reporter enable monitoring mitophagy

**Fig. 4.**
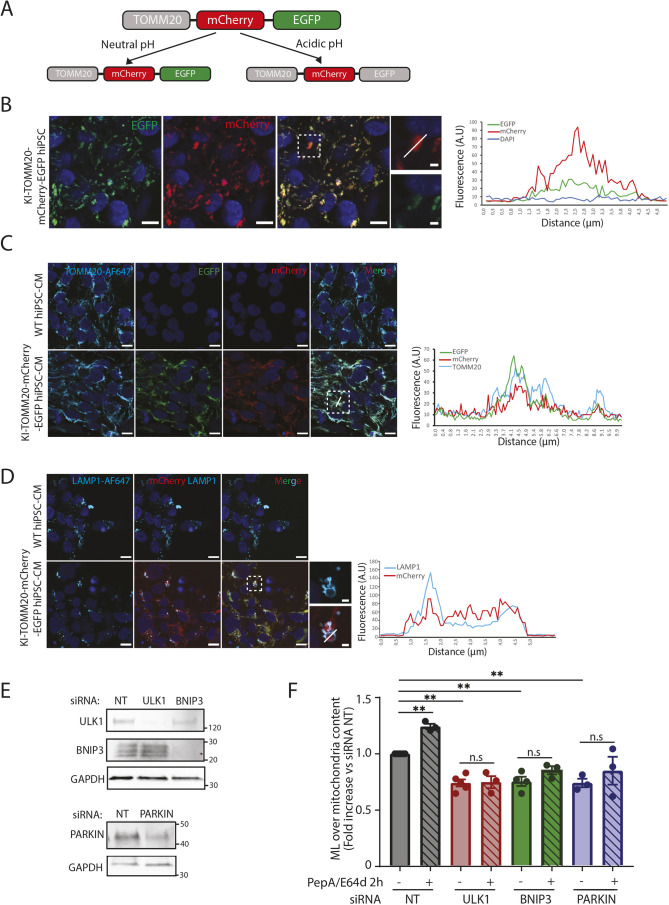
**The TOMM20–mCherry–EGFP pH-sensitive reporter enables monitoring of mitophagy.** (A) Schematic representation of the TOMM20–mCherry–EGFP pH-sensitive reporter. The mCherry and EGFP fluorescent tags were knocked-in after the 3′end of the endogenous *TOMM20* gene by applying CRISPR/Cas9. Red and green fluorescence colocalize in the mitochondria network in neutral pH conditions, displaying yellow fluorescence in merged images. Green fluorescence is quenched in acidic compartments while mCherry remains stable. Red-only structures indicate mitochondria in acidic late endosomes or lysosomes. (B) Representative confocal images of knock-in TOMM20–mCherry–EGFP hiPSCs to verify expression of EGFP (green) and mCherry (red) fluorescence. Representative images of three independent experiments. A red-only dot (acidic mitochondrion) is indicated in the enlarged boxed area and a line profile through the dot is displayed. (C) TOMM20 staining (cyan) was performed in WT and KI-TOMM20–mCherry–EGFP hiPSC-derived cardiomyocytes. Representative confocal images of three independent experiments with cardiomyocytes from a distinct hiPSC differentiation are shown. The line profile for mCherry and EGFP fluorescence intensity and the intensity of TOMM20 staining is displayed. (D) LAMP1 staining (cyan) analysis in WT and KI-TOMM20–mCherry–EGFP hiPSC derived-cardiomyocytes. A red-only dot inside a LAMP1 ring is indicated in the enlarged boxed area and a line profile through the dot is displayed. Representative confocal images of three independent experiments with cardiomyocytes from distinct hiPSC differentiations. (E) Representative western blots displaying the expression level of ULK1, BNIP3 or PARKIN in TOMM20–mCherry–EGFP hiPSC-derived cardiomyocytes for verification of successful siRNA knockdown after 72 h. GADPH is used as a loading control. NT: No target (scramble control siRNA). The data represents three to four independent experiments. (F) Red-only dot quantification in TOMM20–mCherry–EGFP cardiomyocytes transfected with the indicated siRNA for 72 h and treated for the last 2 h with 10 µg/ml PepA and 10 µg/ml E64d lysosomal inhibitors. The number of mitolysosomes (MLs) was quantified and normalized to mitochondrial content using the mito-QC Counter plugin for ImageJ. The data represent three to four independent experiments with cardiomyocytes from distinct hiPSC differentiations. Data are represented as fold increase versus control (NT) as mean±s.e.m. **P<0.01; n.s., not significant (one-way ANOVA with Dunnett's test for multiple comparisons). NT, not transfected; A.U., arbitrary units. Scale bars: 10 µm (B–D, main images); 2 µm (B,D, magnified views).

To directly assess and visualize mitophagy in EHTs, we generated a pH-sensitive mitophagy reporter hiPSC line, by CRISPR/Cas9 mediated knock-in (KI) of a tandem tag of mCherry and EGFP just after the 3′end of the endogenous *TOMM20* gene (KI-TOMM20–mCherry–EGFP). This resulted in the expression of the TOMM20 protein with a C-terminal fusion to the mCherry–EGFP tandem tag (Fig. 4A). With this reporter, the mitochondrial network appears yellow in merged fluorescence images, due to the colocalization of the EGFP and mCherry fluorophores. In acidic compartments, such as late endosomes and lysosomes, the EGFP fluorescence is quenched while the acid-stable mCherry remains fluorescent. Therefore, during imaging, mitochondria or parts of mitochondria in acidic compartments appear as red-only structures in merged images (Shaner et al., 2005). We have recently demonstrated the applicability of a similar reporter for the visualization and assessment of mitophagy in H9c2 cardiomyoblasts (Godtliebsen et al., 2023). Confocal microscopy analysis of KI-TOMM20–mCherry–EGFP hiPSCs showed colocalization of EGFP and mCherry fluorescence and the presence of red-only dots (Fig. 4B). The hiPSC line was carefully validated to ensure the proper function of the mitophagy reporter. Sequencing of the hiPSCs revealed a correct insertion of the tag at the targeted 3′end of the *TOMM20* gene (Fig. S2A). Western blots of the hiPSCs displayed the presence of both the endogenous protein and the reporter tagged TOMM20, indicating that the hiPSCs were heterozygous with respect to the inserted reporter (Fig. S2B). Unfortunately, karyotyping of the hiPSCs revealed trisomy 1 (Fig. S2C). This has been previously reported in culture of the parent (wild type, WT) hiPSC cell line (UKEi003-C) after gene editing (Madsen et al., 2020) and trisomy 1 is a common chromosomal alteration seen in hiPSC clonal selection culture (Närvä et al., 2010; Taapken et al., 2011). Notably, EHTs with trisomy 1 did not display functional differences from WT EHTs (Madsen et al., 2020). Due to the fact that the *TOMM20* gene is on chromosome 1 our reporter hiPSC line thus has overexpression of TOMM20. We still argue that our hiPSC line enables direct assessment of mitophagy given that overexpression of tandem-tagged TOMM20 has previously been used for analysis of mitophagy (Godtliebsen et al., 2023). Immunostaining against the mitochondria inner membrane protein ATP synthase subunit α (ATP5F1A) and TOMM20 confirmed the accurate localization of the tandem tag on mitochondria in hiPSCs (Fig. S2D,E). Finally, we demonstrated that the KI of the tandem tag did not affect the stemness of the hiPSCs that demonstrated high expression (∼98%) of TRA1-60 and SSEA-3 stemness markers (Fig. S2F).

The hiPSCs were successfully differentiated into cardiomyocytes where the mitophagy reporter function was also verified. Cardiomyocyte characteristics were assessed through determining cTnT expression levels by flow cytometry and α-actinin immunostaining (Fig. S2G,H). The TOMM20 immunostaining of the cardiomyocytes revealed colocalization of the tandem tag with TOMM20 on the mitochondria. Moreover, mitochondrial morphology (visualized through TOMM20 staining) was similar to that detected in the WT hiPSC-derived cardiomyocytes (Fig. 4C). Furthermore, LAMP1 immunostaining confirmed that the red-only dots were present inside lysosomes (Fig. 4D). Finally, to uncover mitophagy pathways underlying the generation of acidic (red-only) mitochondria in the cardiomyocytes, we performed siRNA-mediated knockdown of the known mitophagy mediators ULK1, BNIP3 and PARKIN, respectively. Efficient knockdown was confirmed by western blotting (Fig. 4E). To assess mitophagy flux we employed the lysosomal inhibitors PepstatinA and E64d (PepA/E64d) during the last 2 hours of the siRNA knockdown. PepA/E64d treatment does not affect the acidification of lysosomes but blocks cargo degradation (Klionsky et al., 2021) and thus will lead to accumulation of red-only structures if the flux is intact. Interestingly, knockdown of each of these central mitophagy regulators resulted in a significant reduction in the abundance of acidic mitochondria. Furthermore, a lack of an increase in red-only dots in the presence of the lysosomal inhibitors during siRNA knockdown indicates that the mitophagy flux is inhibited (Fig. 4F). Taken together, these results demonstrate that the pH-sensitive reporter enables monitoring of mitophagy in the cardiomyocytes.

### I/R induces mitophagy in EHTs

**Fig. 5.**
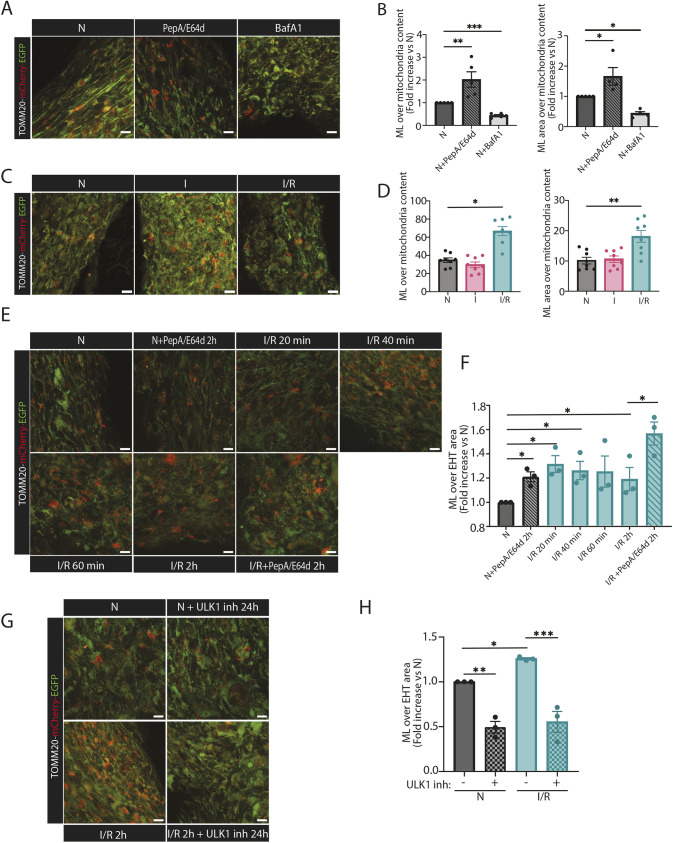
**The TOMM20–mCherry–EGFP mitophagy reporter EHTs reveal induced mitophagy flux during I/R simulation.** (A) Representative confocal images of TOMM20–mCherry–EGFP EHTs subjected to treatment with PepA (10 µg/ml) and E64d (10 µg/ml) or 200 nM BafA1 for 2 h. (B) The number and area of red-only dots, corresponding to mitolysosomes (MLs), were quantified and normalized to mitochondrial content in confocal images for the treatment groups presented in A using the mQC counter plugin for ImageJ. The results are expressed as a fold increase versus control (N). The data represents five independent experiments. (C) Representative confocal images of TOMM20–mCherry–EGFP EHTs subjected to N, I for 90 min and I/R simulation with R for 2 h after I as displayed in Fig. 1A. (D) The number and area of MLs in images of EHTs presented in C was quantified and normalized to mitochondrial content using the mito-QC Counter plugin for ImageJ. The values presented in the graphs are averages from at least eight images (each ROI corresponds to an average of 7000 µm^2^ area) per group in eight independent experiments (a total of at least 64 images per group were analyzed). (E) Representative confocal images of TOMM20–mCherry–EGFP EHTs subjected to N and I/R simulation with a reperfusion time-course including 20 min, 40 min, 60 min and 2 h of reperfusion. The TOMM20–mCherry–EGFP EHTs were also treated with 10 µg/ml PepA and 10 µg/ml E64d for 2 h in the N and in the 2 h I/R simulation groups. The data represent three independent experiments. (F) The number of MLs presented in E was quantified and normalized to EHT area using the mQC counter plugin for ImageJ. The results are expressed as a fold increase versus control (N). The data represent three independent experiments. (G) Representative confocal images of TOMM20–mCherry–EGFP EHTs pre-treated with 1 µM of the ULK1 inhibitor MRT68921 for 24 h and then subjected to N and I/R simulation with R for 2 h after I. The data represent three independent experiments. (H) The number of MLs presented in E was quantified and normalized to EHT area using mQC counter plugin for ImageJ. The results are expressed as a fold increase versus control (N). The data represent three independent experiments. Data are represented as mean±s.e.m. *P<0.05, **P<0.01, ***P<0.005 (one-way ANOVA with Dunnett's test for multiple comparisons). For each independent experiment the EHTs were generated with cardiomyocytes from a distinct hiPSC differentiation. Scale bars: 10 µm.

The KI-TOMM20–mCherry–EGFP cardiomyocytes were subsequently used to generate EHTs. Mitochondrial respiration measurements of the mitophagy reporter EHTs indicated similar function of the mitochondria as compared to WT EHTs (Fig. S3A). To confirm that red-only mitochondria in the EHTs are in an acidic environment within lysosomal structures, EHT sections were immunostained against LAMP1. LAMP1-positive structures colocalized with red-only mitochondria dots, and the red fluorescence was present inside the LAMP1-positive rings (Fig. S3B). Additionally, to validate that the appearance of red-only mitochondria in the EHTs was dependent on low pH inside lysosomes and that the mitochondrial reporter responded dynamically to the lysosomal acidic pH, we treated the EHTs with BafA1 or PepA/E64d, respectively. Red-only structures were quantified, and, as expected, the abundance of mitochondria exhibiting red-only fluorescence decreased in response to BafA1 treatment owing to the loss of acidic pH in lysosomes. By contrast, PepA/E64d treatment of the EHTs led to an increase in the number of red-only dots, demonstrating the dependence of lysosomal cathepsins for degradation of red-only mitochondria (Fig. 5A,B). In addition, to demonstrate the effect of a known mitophagy inducer, we performed carbonyl cyanide-*p*-trifluoromethoxyphenylhydrazone (FCCP) treatment of the EHTs for 24 h with and without PepA/E64d for the last 6 h. As anticipated, FCCP treatment led to an increase in acidic mitochondria and this was further enhanced in the presence of PepA/E64d, revealing induced mitophagy flux (Fig. S3C,D). These results underscore the feasibility of our pH-sensitive mitophagy reporter to study mitophagy flux in EHTs.

To investigate alterations in mitophagy in a physiologically relevant setting, the mitophagy reporter EHTs were subjected to I and I/R as described in Fig. 1A. Fixed whole EHTs or sections were then imaged and the acidic mitochondria quantified. Under control conditions, red-only mitochondria structures were detected, revealing a basal level of mitophagy in EHTs. During I, there was no detectable change in the number of red-only structures compared to the control. Intriguingly, there was a significant increase in red-only structures in EHTs after I/R (Fig. 5C,D; Fig.S4A). To address the kinetics and the extent of mitochondrial degradation during reperfusion in the EHTs, we performed a time-course experiment with 20, 40 and 60 min of reperfusion in addition to the original 2 h reperfusion timepoint. For flux assessment, we included PepA/E64d during 2 h of reperfusion. The results indicate a rapid increase in acidic mitochondria after only 20 min of reperfusion. After 2 h of reperfusion, the level was slightly lower than at 20 min, but in the presence of PepA/E64d the abundance of acidic mitochondria was significantly increased, demonstrating an induction of mitophagy flux (Fig. 5E,F). Western blot of the TOMM20–mCherry–EGFP fusion protein in EHTs during a I/R time-course experiment also revealed a reduction in the level of the fusion protein during reperfusion, in accordance with an enhanced degradation (Fig. S4B). To investigate the involvement of ULK1 in induced mitophagy during I/R simulation in the EHTs, we performed pretreatment of the EHTs with the ULK1 inhibitor MRT68921 (Petherick et al., 2015) for 24 h prior to I/R simulation (ischemia for 90 min and 2 h of reperfusion). Our results demonstrate a significant reduction in the abundance of acidic mitochondria in the presence of the ULK1 inhibitor, both during normal culture conditions and after I/R simulation (Fig. 5G,H). Hence, our data indicate that ULK1 activity is needed for induced mitophagy during I/R. In summary, by employing novel mitophagy reporter EHTs, our results strongly indicate, for the first time, an induction of mitophagy flux during I/R in a human cardiac tissue model.

## DISCUSSION

In this study, we investigated the effect of I/R simulation on mitochondria ultrastructure, function and lysosomal degradation of mitochondria in human EHTs. Our findings revealed impaired mitochondria with a significant decrease in mitochondrial respiration and ATP production following I and I/R simulation. We were able to successfully monitor lysosomal degradation of mitochondria within EHTs, exploiting endogenously labelled (mCherry–EGFP) TOMM20 as a reporter. Here, we detected an induction of mitophagy flux after I/R simulation and ULK1 activity was found to be important for the induction. Our results provide valuable insights into the level of basal mitophagy and alterations during I/R in a human heart tissue model, which is of importance for the therapeutic targeting of mitophagy in the human heart.

Currently, there is a lack of effective treatments to adequately mitigate reperfusion injury in patients (Lesnefsky et al., 2017). A very limited number of preclinically identified promising cardioprotective interventions have been successfully translated to patients (Hausenloy et al., 2017). This can be attributed to the inherent physiological disparities between animal models and humans. Thus, there is a need to establish more reliable models of the human heart. EHTs have the potential to bridge the gap between animal models and humans. Our results demonstrated an increase in cTnI and LDH release from EHTs into the culture medium, indicating cell death. Furthermore, we detected cTnT sarcomere disarrangement after I and I/R simulation. Sarcomere disarrangement following I/R injury is also previously reported in hiPSC-derived cardiomyocytes (Häkli et al., 2021) and EHTs (Funcke et al., 2020). I/R simulation in EHTs thus provides a valid model for studying I/R injury.

Preclinical studies in various animal models, including mice, guinea pigs, dogs and pigs (Heusch et al., 2011; Kajiyama et al., 1987; Szczepanek et al., 2015; Yang et al., 2014) report mitochondrial damage during I/R (Lesnefsky et al., 2017). Specifically, the electron transport chain in mitochondria undergoes progressive injury during I/R, primarily affecting complex II (Borutaite et al., 2001; Heusch et al., 2011; Rouslin, 1983). Conversely, there is a lack of comprehensive investigation of mitochondrial respiration in human heart models. A decrease in mitochondrial respiration during hypoxia (1% oxygen) and increased susceptibility to hypoxia has been demonstrated in 2D hiPSC-derived cardiomyocytes (Peters et al., 2022). However, analysis of mitochondria respiration has not been previously conducted in EHTs in control conditions nor during I/R. In this study, we measured mitochondrial respiration in whole EHTs. Our results are consistent with I/R experiments conducted in animals, revealing a reduction in mitochondrial respiration and ATP production, particularly affecting complex II.

Mitochondria quality control mechanisms in I and I/R remain elusive in the human heart. Mitophagy plays an important role in maintaining cardiac tissue function in response to stress (Morales et al., 2020). However, excessive activation provokes a catastrophic loss of mitochondria (Chen and Dorn, 2013), whereas an insufficient response leads to accumulation of dysfunctional mitochondria, limiting the ability of cardiomyocytes to adapt to stress (Song et al., 2014). Notably, there are contradicting results regarding mitophagy alterations during myocardial I/R in studies with traditional cell lines or animal models. Applying pH-sensitive fluorescent mitochondrial reporters such as mtKeima and *mito*QC enables assessment of mitophagy in animal tissues (Cornelissen et al., 2018; Jiménez-Loygorri et al., 2024; Lee et al., 2018; Mcwilliams et al., 2018, 2016; Munson et al., 2021; Rappe et al., 2024; Singh et al., 2021; Sun et al., 2015). However, reports on the impact of myocardial I/R on mitophagy in these animal models are scarce (Saito et al., 2019). A consensus and relevance to the human heart is essential for therapeutic targeting of mitophagy. Interestingly, there also seems to be a lack of studies that assess mitophagy flux in an I/R setting in hiPSC-derived cardiomyocytes, EHTs or other 3D human cardiac tissue models. Expression of mtKeima in hiPSC-derived cardiomyocytes in 2D culture indicates induction of mitophagy after 6 h of hypoxia (Yang et al., 2022). However, our results indicated no change in lysosomal degradation of mitochondria in EHTs after 90 min of simulated ischemia. Conversely, only 20 min of simulated reperfusion after ischemia increased the level of acidic mitochondria in the mitophagy reporter EHTs. Furthermore, a significant increase was detected after 2 h of reperfusion in the presence of PepA/E64d compared to without, indicative of an induced mitophagy flux. The lysosomal inhibitors PepA/E64d have previously been applied to demonstrate mitophagy flux alterations in cardiac cells expressing dual fluorescent pH-sensitive mitochondrial reporters (Godtliebsen et al., 2023; Kobayashi et al., 2020a,b). Furthermore, the protease inhibitor leupeptin has been recently applied in *mito*QC mice to verify induced mitophagy flux in the retina of aged mice (Jiménez-Loygorri et al., 2024). It is noteworthy that the pH-sensitive reporters themselves do not indicate the mechanism by which the mitochondria or parts of mitochondria arrive into lysosomes. Our siRNA knockdown experiment in the tandem-tagged hiPSC-derived cardiomyocytes implicates ULK1, BNIP3 and PARKIN as being involved in acidification of mitochondria. This implies that parallel mitophagy mechanisms with functional redundancy are at play to ensure degradation of mitochondria and this is consistent with our previous data in H9c2 cardiomyoblasts (Godtliebsen et al., 2023). Furthermore, we cannot exclude the involvement of alternative mitolysosomal pathways that do not depend upon all of the conventional autophagy machinery (Ganley and Simonsen, 2022). In light of the siRNA knockdown results, we subjected the mitophagy reporter EHTs to a pretreatment with a ULK1 inhibitor and demonstrated reliance on ULK1 activity for induced mitophagy flux during I/R. To our knowledge, induction of mitophagy flux after I/R has not previously been demonstrated in human EHT. The importance of ULK1 is not surprising, given the role of ULK1 in autophagy induction and involvement in several mitophagy pathways. Comprehensive deciphering of the underlying mechanisms and revealing whether further induction or inhibition of mitophagy flux after I/R is cardioprotective will be important future tasks. Moreover, our mitophagy reporter EHTs offer new opportunities for studying mitophagy in various pathologically relevant settings for the human heart.

## MATERIALS AND METHODS

### Culture of hiPSCs

The hiPSC line UKEi003-C (Sendai virus reprogrammed) was employed. The cell line is registered at the European Human Pluripotent Stem Cell Registry (https://hpscreg.eu/). The hiPSCs were cultured in mTeSR1 medium (STEMCELL technologies, 85850) in cell culture plates or flasks pre-coated with Geltrex [Gibco, A1413301; 1:100 in RPMI 1640 (Gibco, 21875-34) at room temperature (RT), overnight] in 5% CO_2_ and 5% O_2_. The cells were passaged and cultured according to published protocols (Shibamiya et al., 2020).

### Generation of mitophagy reporter hiPSC line by CRISPR/Cas9

The hiPSCs were genetically modified to insert the pH-sensitive tandem fluorescent reporter tag (mCherry-EGFP) at the 3′ end of the translocase of the outer mitochondrial membrane 20 (*TOMM20*) gene applying a previously described CRISPR/Cas9 knock-in strategy for fluorescent tagging of endogenous *TOMM20* (Roberts et al., 2017). The knock-in donor plasmid AICSP-8:TOMM20-mEGFP was Addgene plasmid #87423 (deposited by Allen Institute for Cell Science). The mCherry tag was inserted into the BamHI restriction site, 5′ of the mEGFP tag of the donor plasmid by In-Fusion cloning (Takara Bioscience; #638948) resulting in the tandem mCherry–EGFP tag. The sgRNA sequence (5′-AATTGTAAGTGCTCAGAGCT-3′, Thermo Fisher Scientific) was cloned into the CRISPR/Cas9 plasmid PX459. This plasmid, pSpCas9(BB)-2A-Puro (PX459) V2.0, was Addgene plasmid #62988 (deposited by Feng Zhang; Ran et al., 2013). The hiPSCs were dissociated using Accutase (Sigma-Aldrich, A6964) and 10^6^ cells were co-transfected with 2 µg of the donor plasmid and the sgRNA plasmid by electroporation using a Neon Transfection System (Thermo Fisher Scientific; #MPK10096). Electroporation was performed with a single pulse at 1300 V for 30 ms and the cells were plated in Geltrex-coated six-well plates and cultured for 2 days. The hiPSCs were dissociated, and cells expressing mCherry and EGFP were sorted using BD FACS Aria™ III cell sorter (BD Biosciences) to obtain single-cell clones in 96-well plates. The cells were kept for 2 days with mTeSR1 medium supplemented with CloneR™ (STEMCELL Technologies, 05888). Clones expressing the mCherry–EGFP tag on TOMM20 were confirmed by microscopy. Positive clones were further expanded and verified by genomic DNA PCR amplification (Fwd primer, 5′-GAATAGCGTGTCTGTTACAAGTGTTG-3′ and Rev primer, 5′-GTCCCACCTGCTCCACTCTTTTC-3′), Sanger sequencing and western blotting.

### Differentiation of hiPSC into cardiomyocytes

For hiPSC differentiation into cardiomyocytes, the cells were seeded in six-well plates coated with Matrigel^®^ (Corning, 354230; 1:60 dilution in DMEM) at a density of 350,000 cells per well. When the cells reached 60% confluence, a 2D-monolayer cardiac differentiation protocol was initiated to first activate and subsequently to inhibit the Wnt signaling pathway by sequential application of growth factors and small molecules (Lian et al., 2012; Mosqueira et al., 2018). Briefly, Matrigel in StemPro-34 medium (Gibco, 11879-020) with 1 ng/ml BMP4 (R&D systems, 314-BP) and L-glutamine (Sigma-Aldrich, G7513) was overlaid on the hiPSCs, and cells were incubated for 12–15 h. The medium was then changed to StemPro-34 containing 8 ng/ml activin A (R&D systems, 338-AC) and 10 ng/ml BMP4 for 48 h. Next, the Wnt pathway was inhibited with 10 µM KY02111 (Tocris; 4730) and 10 µM XAV939 (Tocris, 3748) in RPMI 1640 with B27 without insulin (1:50; Sigma-Aldrich; A1895602) for another 48 h. Finally, the medium was changed to RPMI 1640 supplemented with B27 with insulin (Sigma-Aldrich; 17504044) and KY0211 and XAV939 for the final 48 h. Thereafter, the cells were fed every other day with RPMI 1640 containing B27 with insulin and monitored for spontaneous beating (day 8–10). When coherent beating of the cardiomyocytes was achieved (usually around day 14–15) the cardiomyocytes were gently dissociated.

### Dissociation of cardiomyocytes

The hiPSC-derived cardiomyocytes were dissociated using a collagenase-based approach. The cardiomyocytes were washed twice with Ca^2+^ and Mg^2+^-free Hank's balanced salt solution (HBSS; Life Technologies, 14175095) and subsequently incubated with 1 mg/ml collagenase II (200 U/ml; Worthington, LS004176) in Ca^2+^/Mg^2+^-free HBSS, supplemented with 10 mM HEPES (Sigma-Aldrich, H4034), 10 μM Y-27632 (Biorbyt, orb154626) and 30 μM N-benzyl-*p*-toluenesulfonamide (BTS, TCI, B3082), for 2–4 h at 37°C at 5% CO_2_. Dissociated cardiomyocytes were then collected from the flasks and washed with RPMI 1640 supplemented with 6 μg/ml deoxyribonuclease II (DNAseII; Sigma-Aldrich, D8764), followed by centrifugation at 100 ***g*** for 10 min. Thereafter, the cells were resuspended in warm RPMI 1640 containing 10 μM Y-27632 and 1% penicillin-streptomycin (P/S; Sigma-Aldrich, P4333), counted using an automated cell counter (Invitrogen, Countess II).

### siRNA transfection of cardiomyocytes

The siRNAs used were pre-designed and validated *Silencer*^®^Select siRNAs (Invitrogen, 4392420): siRNA against *ULK1* (siRNA ID s15963), siRNA against *BNIP3* (siRNA ID s2059) and siRNA against *PARKIN* (siRNA ID s502575). The hiPSC-derived cardiomyocytes were plated in eight-well chambered cover glass (Cellvis, C8SB-1.5H) or 12-well plates coated with Geltrex. When the hiPSC-derived cardiomyocytes had resumed the beating after plating, they were transfected with siRNA using Lipofectamine RNAiMax Transfection Reagent (Invitrogen, 13778-075) according to the manufacturer's recommendation. After 16 h of incubation, the medium was changed to remove the transfection reagent. The cells were treated with 10 µg/ml Pep A and 10 µg/ml E64d for the final 2 h of 72 h siRNA knockdown (Table S1). The cells were consequently fixed at 37°C for 20 min with Roti®-Histofix (Roth, P087.6) for red-only dot analysis or harvested for western blot analysis. For each condition, 10 positions were selected and imaged as a Z-stack.

### Flow cytometry

Single-cell suspensions of hiPSCs or hiPSC-derived cardiomyocytes were analyzed by flow cytometry. The hiPSCs were analyzed for pluripotent stemness markers. Briefly, the hiPSCs were harvested and resuspended in blocking buffer containing 5% FBS (Sigma-Aldrich, F2442) in PBS for 15 min at 4°C and stained against SSEA-3 and TRA1-60 and the corresponding isotype controls (Table S2) for 30 min at 4°C. Then, the cells were washed with PBS and fixed with cold methanol for 20 min at 4°C followed by a final washing step with PBS. The hiPSC-derived cardiomyocytes were analyzed for the cardiac marker cTnT. The cells were first stained with Live/Dead staining reagent (Invitrogen; 65-0863-14) in 5% FBS for 30 min at 4°C. The cells were subsequently fixed in Roti®-Histofix (Roth, P087) for 20 min at 4°C, blocked and permeabilized in FACS buffer containing 5% FBS, 0.5% Saponin (Sigma-Aldrich, S7900) and 0.05% sodium azide (Sigma-Aldrich, S8032) in PBS. Cells were stained against cTnT and the corresponding isotype control (Table S2) and washed with PBS. Samples were analyzed with the BD LSRFortessa™ flow cytometer and the BDFacsDiva™ software. Cells were selected by gating SSC-A versus FSC-A and gate for fluorescence intensity using isotype-stained cells as controls.

### Karyotyping

A successfully genetically modified clone of KI-TOMM20–mCherry–EGFP hiPSCs was subjected to karyotype analysis using KaryoStat+ Genetic Stability Assay (Thermo Fisher Scientific; A52849). Briefly, 2×10^6^ hiPSCs were dissociated and washed with PBS. The cells were centrifuged for 3 min at 500 ***g*** and the pellet was flash-frozen with liquid nitrogen and kept at −80°C until the sample was ready to ship for further analysis by Thermo Fisher Scientific.

### EHT

EHTs were generated from hiPSC-derived cardiomyocytes and fibrinogen/thrombin (Sigma-Aldrich, F8630 and 605157) hydrogels in polydimethylsiloxane (PMDS) racks as previously described (Breckwoldt et al., 2017). Casting molds were prepared by placing the Teflon spacer in 24-well culture plates and adding 2% agarose (Invitrogen, 16500500) in PBS. After agarose solidification, the spacer was removed and the PMDS silicon racks were placed inside the casting molds. Dissociated WT or KI-TOMM20–mCherry–EGFP hiPSC-derived cardiomyocytes were resuspended in DMEM (Biochrom; F0415) containing 1% P/S (Sigma-Aldrich, P4333), 10% heat-inactivated horse serum (HS; Gibco, 26050088), 1% L-glutamine, 0.1% Y-27632 and 0.5 µg/mg fibrinogen (stock solution: 200 mg/ml plus aprotinin 0.5 µg/mg fibrinogen in NaCl 0.9%, Sigma-Aldrich, F4753). The cell concentration was adjusted to 10×10^6^ cells/ml. For each EHT, 100 µl of the cell suspension (10^6^ cells) were mixed with 3 µl thrombin (100 U/ml; Sigma-Aldrich, T7513) and pipetted into the agarose mold containing the PMDS rack. For fibrinogen polymerization, the constructs were placed in a 37°C, 5% CO_2_ and 40% O_2_ humidified cell culture incubator for 90 min. Cell culture medium was subsequently added to the wells to ease removal of the EHT constructs from the agarose mold. The racks with the EHTs were then transferred to a new 24-well cell culture plate. EHT were maintained in a 37°C, 5% CO_2_ and 40% O_2_ humidified cell culture incubator. Medium was changed on every other day. EHT medium consisting of DMEM, 10% heat-inactivated HS, 1% P/S, 10 µg/ml insulin (Sigma-Aldrich I9278), 400 µM tranexamic acid (Sigma-Aldrich, 857653) and 33 µg/ml aprotinin (Sigma Aldrich A1153). EHTs were monitored for spontaneous and coherent beating deflecting the silicon posts, usually occurring at ∼5–7 days after casting. EHTs were allowed to mature for 3 weeks and then the experiments were performed.

### Ischemia and ischemia/reperfusion simulation

To simulate ischemia (I), the EHTs were incubated for 90 min in glucose free DMEM (Gibco, A14430-01) supplemented with 4 mM sodium DL lactate (Sigma-Aldrich; L4263), 10 µg/ml insulin, 33 µg/ml aprotinin and 1% P/S (Funcke et al., 2020) in hypoxic conditions (0.3% O_2_). Before performing I, the medium was kept overnight in the hypoxia chamber to remove all oxygen in the medium. The EHTs were washed twice with PBS prior to I simulation. For reperfusion (R) simulation, the EHTs were placed back to their complete culture medium in a cell culture incubator with 40% O_2_.

### EHT fixation, vibratome sectioning and immunostaining

EHTs were fixed with Roti^®^-Histofix for 16 h at 4°C and subsequently embedded in 4% agarose for vibratome (Leica, VT1200S) sectioning into 70 µm sections. For red-only dot quantification, EHT sections or whole EHTs were stained with DAPI (1:1000; Thermo Fisher Scientific, 62248) for 30 min. The sections or EHTs were washed three times with PBS and mounted on glass slides using Prolong Glass Antifade Mountant (Invitrogen, P36980) and #1.5 coverslips (Zeiss, 4740309000). For immunostaining, the EHT sections were blocked and permeabilized in 0.05 M Tris-buffered saline (TBS) containing 10% fetal bovine serum (FBS; Sigma-Aldrich, F2442), 1% bovine serum albumin (BSA; Sigma-Aldrich, A7030) and 0.5% Triton™X-100 (Sigma-Aldrich; 93443) for 90 min at room temperature. The sections were washed and incubated with primary antibody (Table S2) diluted in antibody solution (0.05 M TBS, 1% BSA and 0.5% Triton X-100) overnight at 4°C. The EHT sections were then washed three times with PBS and incubated with a Highly Cross-adsorbed Alexa Fluor-coupled secondary antibody (Table S2) and DAPI in antibody solution for 90 min at room temperature. Finally, the EHT sections were washed five times with PBS and mounted on glass slides (Epredia, AA00000112E01MNZ10) using Prolong Glass Antifade Mountant and #1.5 glass coverslips.

### Proximity ligation assay

For PLA or DuoLink, the EHT sections were placed on Superfrost™ Plus Adhesion Microscope slides (Epredia; J1800AMNZ) and air-dried for 20 min to allow the EHT sections to adhere to the slides. Duolink (Sigma-Aldrich, Duo9200) was performed according to the manufacturer's instructions in a slide incubator chamber (Epredia, 73310017) to minimize the volume of the reagents. Briefly, EHT sections were permeabilized and blocked for 90 min at room temperature in the same blocking solution as used for immunostaining (0.05 M TBS, 1% BSA, 10% FBS and 0.5% Triton X-100) and then incubated overnight with the primary antibodies against TOMM20 and LAMP1 in antibody solution (0.05 M TBS, 1% BSA and 0.5% Triton X-100) at 4°C. As a negative control, one section was incubated with only one primary antibody (Table S2). The sections were washed and then incubated with the corresponding PLA probes Anti-Rabbit Plus (Sigma-Aldrich; DUO92002) and Anti-Mouse Minus (Sigma-Aldrich; DUO92101) for 1 h. Subsequently, the EHT sections were washed and incubated with the ligation solution for 1 h, followed by incubation with amplification far-red solution (Sigma-Aldrich, Duo92013) for 2 h. All incubation steps were performed in the humidified slide incubator chamber at 37°C. Finally, the EHT sections were washed and stained with DAPI, air-dried for 20 min, and mounted with Prolong Glass Antifade Mountant.

### Western blotting

For western blot analysis of hiPSC-derived cardiomyocytes, the cells were washed twice with PBS and lysed by scraping in 2× SDS buffer (100 mM Tris-HCl, pH 6.8, 20% glycerol and 4% SDS) and boiling for 5 min. For EHT western blot analysis, three EHTs per group were pooled together, flash-frozen with liquid nitrogen, and homogenized using T-PER buffer (Thermo Fisher Scientific, 78510) supplemented with 1× Complete™ Mini EDTA-Free Protease Inhibitor Cocktail (Roche, 11697498001) and phosphatase inhibitors (Calbiochem, 524625) and boiled in SDS loading buffer for 10 min at 95°C. Protein concentrations were determined using a bicinchoninic acid assay (Thermo Fisher Scientific; 23225) and 20–30 µg of total protein lysates were run on Mini-Protean® TGX™ precast 4–20% gradient gels (Bio-Rad, 456-1093) and transferred onto Invitrolon™ PVDF membranes (Invitrogen, LC2005). The transfer was visualized with Ponceau staining (Thermo Fisher Scienctific; A40000278) and the membrane was blocked with 5% non-fat dry milk (Sigma-Aldrich, 70166) in TBST (Sigma-Aldrich, P1379). The membrane was incubated with primary antibody (Table S2) overnight at 4°C followed by 1 h incubation at room temperature with horseradish peroxidase (HRP)-conjugated secondary antibody; BD Pharmingen HRP-conjugated goat anti-mouse-IgG (BD Biosciences, 554002) or HRP-conjugated Affinipure goat anti-rabbit-IgG (H+L) (Proteintech, SA00001-2). Signal detection was performed with a western blotting chemiluminescent reagent (Sigma-Aldrich, CPS3500) and an iBright Imaging System (Thermo Fisher Scientific). Uncropped images of western blots from this paper are shown in Fig. S5.

### Lactate dehydrogenase analysis

Cell death was analyzed through LDH release into the medium. LDH quantification of the medium was performed using CyQuant™ LDH cytotoxicity assay (Sigma-Aldrich, C20300) with a modified protocol. Briefly, EHT medium was collected after each treatment and centrifuged at 200 ***g*** to remove cell debris. A total of 50 µl of EHT medium was mixed with 50 µl of the reaction buffer according to the manufacturer's instruction and incubated for 30 min at room temperature in triplicates. The reaction was stopped with Stop Solution and absorbance was measured at 490 nm and 680 nm (background signal) using SpectraMax®ID3 (Molecular Devices). Medium not containing EHT was also analyzed and the measured value was subtracted from the measured sample values.

### cTnI analysis

EHT culture medium was collected after every treatment according to Fig. 1A and was frozen immediately after centrifugation for 10 min (200 ***g***, room temperature) and stored at −70°C until subsequent analysis. The amount of cTnI in the medium was quantified in duplicates from four different experiments using a bead-based multiplex assay (Bioplex; Biorad Laboratories, Hercules, Calif). Final cTnI concentrations were calculated using the Bioplex software supplied by the manufacturer.

### ATP analysis

ATP production in the EHTs was analyzed using CellTiter-Glo^®^ Assay (Promega, G7570). The cellTiter-Glo® reagent was prepared according to the manufacturer's instructions and diluted 1:1 with PBS. A total of 100 µl of the solution was pipetted into opaque-walled 96 multiwell plates (Thermo Fisher Scientific; 1502). EHTs were removed from their silicon posts and incubated with the solution for 5 min on an orbital shaker to induce cell lysis, followed by 20 min incubation on the bench at room temperature to stabilize the luminescent signal. Luminescence was recorded for two EHTs per condition in each experiment according to the manufacturer's instructions using a SpectraMax®ID3 (Molecular Devices).

### Mitochondrial respiration

Respirometry was performed in an Oxygraph-2k system (Oroboros Instruments, Innsbruck, Austria) calibrated to air (gain for oxygen sensor was set to 2) with mitochondrial respiration medium (110 mM sucrose, 60 mM K+-lactobionate, 0.5 mM EGTA, 3 mM MgCl_2_, 20 mM taurine, 10 mM KH_2_PO_4_, 20 mM HEPES pH 7.1) at 37°C. In total, two EHTs by condition were removed from the silicon rack and inserted into the Oxygraph chamber. The chambers were sealed to obtain a closed system. Analysis of the oxygen concentration in the chambers was performed using DatLab version 5.1.0.20 (Oroboros Instruments, Innsbruck, Austria). Decreasing oxygen concentration in the chambers resembled cellular oxygen consumption. When the oxygen consumption rate, OCR [O2 flux (pmolO_2_/s·ml)] reached a steady state level, a measurement was recorded displaying total cellular respiration (basal). Digitonin was added to permeabilize the cardiomyocytes within the EHTs. A final concentration of 0.1 mM malate (Sigma-Aldrich; M1000) and 5 mM pyruvate (Sigma-Aldrich; P2256) served as substrates for complex I. *V*_0_ was defined as the respiration in the presence of substrates before ADP was added. OXPHOS capacity was assessed by the addition of 2.5 mM ADP (Merck-Calbiochem; 117105-1GM). Mitochondrial outer membrane integrity was measured after adding 2.5 mM cytochrome *c* (Sigma-Aldrich; C7752). A concentration of 100 mM succinate (Sigma-Aldrich; S2378) was added to assess the complex II efficiency. Subsequently, the proton gradient was released by stepwise titration (0.5 μM/step) of the uncoupler carbonyl cyanide-*p*-trifluoromethoxyphenylhydrazone (FCCP) (Sigma-Aldrich, C2920) until the maximum respiration was achieved (electron transport system capacity, ETS capacity). Then, 0.5 µM rotenone (Sigma-Aldrich; R8875) was added to inhibit complex I function. Finally, 2.5 μM antimycin A (Sigma-Aldrich, A8674) an inhibitor of complex III blocked mitochondrial respiration completely, resulting in the residual oxygen consumption (ROX).

### Confocal imaging and imaging analysis

For confocal imaging, a LSM800 microscope (Carl Zeiss Microscopy) was used with a Plan-Apochromat 63× oil (M27) objective with an NA of 1.4 and LSM900 microscope (Carl Zeiss Microscopy) was used with a Plan-Apochromat 40× oil (M27) objective with an NA of 0.95. All images were acquired with z-stacks. For red-only dot/mitolysosome (ML) quantification, a mito-QC Counter macro for FIJI/ImageJ was used (Montava-Garriga et al., 2020; https://github.com/graemeball/mQC_counter). Here, it is the ratio of red fluorescence over green fluorescence that influences the ML count which then is normalized against total mitochondrial content per image. The parameter ‘ML over mitochondrial content’ (‘nML_over_green’ in the macro measurements) reflects the number of detected red-only dots/mitolysosomes (MLs) divided by green mean intensity (mitochondrial content) per image. ‘ML area over mitochondrial content’ (‘MLarea_μm2_over_green’ in the macro measurements) reflects the total measured area of all the ML/red only dots divided by green mean intensity (mitochondrial content) per image. Here, for both 2D cardiomyocytes and EHTs, *z*-stacks were collapsed with ImageJ. For the 2D cardiomyocytes, the region of interest (ROI) was defined as the entire image frame. For the EHTs, the ROI was selected for the area containing elongated cells, situated at the outer edges of the EHTs. Mitolysosomes/mitochondria_content and Mitolysosomes_area/mitochondria content were analyzed and quantified per image. For LAMP1 staining area quantification, a Volocity^®^-based cell quantification was used. Confocal images were imported into Volocity^®^ in a tiff file format. ROIs were selected for areas with elongated cells situated along the edges of the EHT. The ‘find objects’ command was used and adjusted to select LAMP1 rings. Settings were maintained between all conditions.

### Processing of EHTs for TEM

EHTs attached to silicon posts were fixed by adding two volumes of pre-warmed, double-strength fixative to each well, and left for 1 h at room temperature. The final fixative concentration was 4% formaldehyde and 1% glutaraldehyde in PHEM buffer (60 mM PIPES, 25 mM HEPES, 10 mM EGTA, 4 mM MgSO_4_·7H_2_O, pH 7.4). The tissue was then rinsed twice in PBS, carefully released from the posts using a scalpel, and transferred to a glass vial with PBS. Subsequent fixation, contrasting and dehydration steps were carried out in a microwave processor (Pelco BioWave, Ted Pella, Inc.). Tissues were post-fixed in 4% formaldehyde, 1% glutaraldehyde and 0.05% Malachite Green (Sigma-Aldrich, 101398) in PHEM buffer, followed by 1% osmium tetroxide (EMS, 19110) and 0.8% K_3_Fe(CN)_6_ (Sigma-Aldrich, 702587) in 0.2 M sodium cacodylate buffer (pH 7.4), then followed by 1% tannic acid (EMS, 21700) in ddH_2_O, and finally in 1% uranyl acetate (EMS, 22400) in ddH_2_O. Several washes with buffer or ddH_2_O were performed between steps to prevent reagent carry over. The tissue was then dehydrated in a graded acetone series (30%, 60%, and 90%) and three changes of pure acetone, followed by five changes of pure acetone (5 min each) on the bench. Finally, the tissue was embedded in epoxy resin (Agar, R1043) as follows: acetone was first replaced with a 3:1 mixture of acetone and resin (without accelerator) for 1 h, then with a 1:1 mixture overnight. The following day, the 1:1 mixture was replaced with three changes of pure resin (with accelerator), left overnight at room temperature, and then set to polymerize at 60°C for 48 h.

### Trimming and ultrathin sectioning

After polymerization, excess resin around the tissue was removed using a razor blade, and an EM TRIM2 diamond miller (Leica Microsystems) was used to expose an area of interest on the EHT and trim the block for ultramicrotomy. The block was mounted on a UC6 ultramicrotome (Leica Microsystems) equipped with a 35° Ultra diamond knife (Diatome). A series of 60 nm ultrathin sections were cut and collected on 3 mm formvar-coated slot grids.

### TEM

Ultrathin sections were imaged on an HT7800 transmission electron microscope (Hitachi High-Tech) operated in high contrast mode at 100 kV, using a Xarosa CMOS camera with Radius ver. 2.0 software (EMSIS). For each experimental condition, areas of tissue with cells showing clear characteristics of cardiomyocyte differentiation (myofibrils, sarcomeres, and intercalating discs) were identified and imaged at low (2000×), intermediate (7000×) and high (15,000×–40,000×) magnification.

### Statistical analysis

Statistical analysis was performed using GraphPad Prism version 8.0.1 for Windows (GraphPad Software, San Diego, California USA). All results are expressed as mean±standard error of the mean (s.e.m.). Groups were compared using one-way ANOVA with Dunnett's test for multiple comparisons. At least three different independent differentiation batches were used. The exact number of biological replicates for each experiment and *P*-values are indicated in the figure legends.

## Supplementary Material



10.1242/joces.263408_sup1 Supplementary information
