# Supplementary material for: Mitophagy is induced in human engineered heart tissue after simulated ischemia and reperfusion
Source: J Cell Sci. 2025 Mar 19;138(9):jcs263408. doi: 10.1242/jcs.263408 (PMC11959618; doi:10.1242/jcs.263408)
Supplement: Supplementary information [file joces-138-263408-s1.pdf]

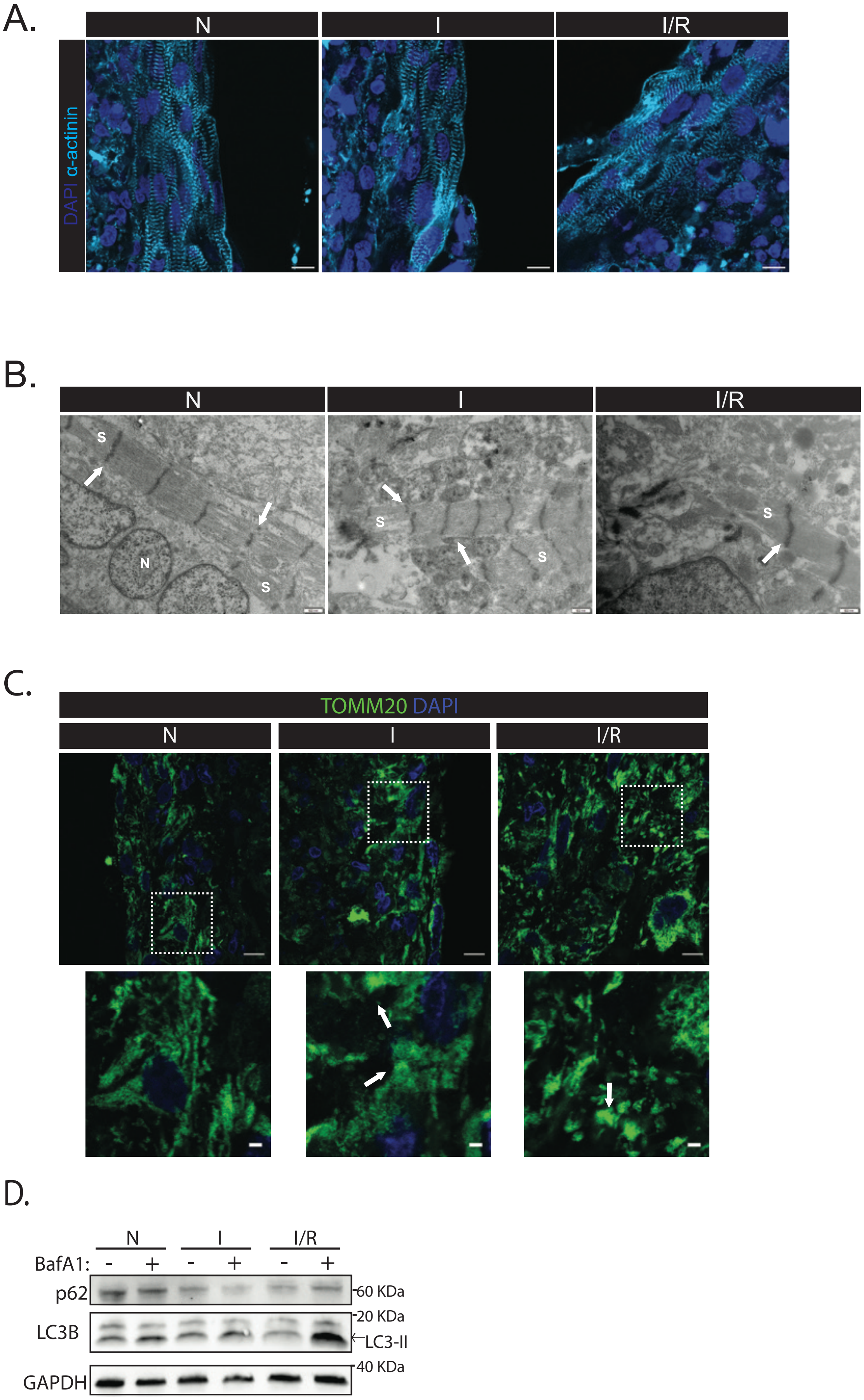
A.
N
I
I/R
DAPI α-actinin
B.
N
I
I/R
S
N
C.
TOMM20 DAPI
N
I
I/R
D.
N
I
I/R
BafA1:
- + - + - +
p62
LC3B
GAPDH
60 KDa
20 KDa
LC3-II
40 KDa

Fig. S1. Sarcomere visualization and changes in mitochondrial morphology during I and I/R simulation.

A. EHTs were subjected to N, I for 90 min and I/R for 2 h, immunostained against α-actinin (cyan) and stained with DAPI (blue). Representative images of four independent experiments.

B. TEM images of EHTs in N, I for 90 min and I/R for 2 h reveal sarcomere organization. S: sarcomeres, N: nucleus. White arrows indicate the z-lines in the sarcomeres. Representative images of two independent experiments.

C. TOMM20 immunostaining (green) and DAPI (blue) staining in sections from EHTs subjected to N, I for 90 min and I/R for 2 h. Insets show magnified views of boxed areas indicated in the top images. Arrows indicate more rounded and clustered mitochondria. Representative confocal images of three independent experiments.

D. Representative western blot analysis of the expression of the autophagy markers LC3 and p62 in total cell lysates from homogenized EHTs subjected to N, I for 90 min and I/R for 2 h in the presence of BafA1 (+) or not (-). The lipidated form of LC3B (LC3-II) is indicated with an arrow. The data represent three independent experiments.

Data information. For each independent experiment the EHTs were generated with cardiomyocytes from a distinct hiPSC differentiation. Scale bar: 10 µm (A, C), 2 µm (insets in C) and 500 nm (B).

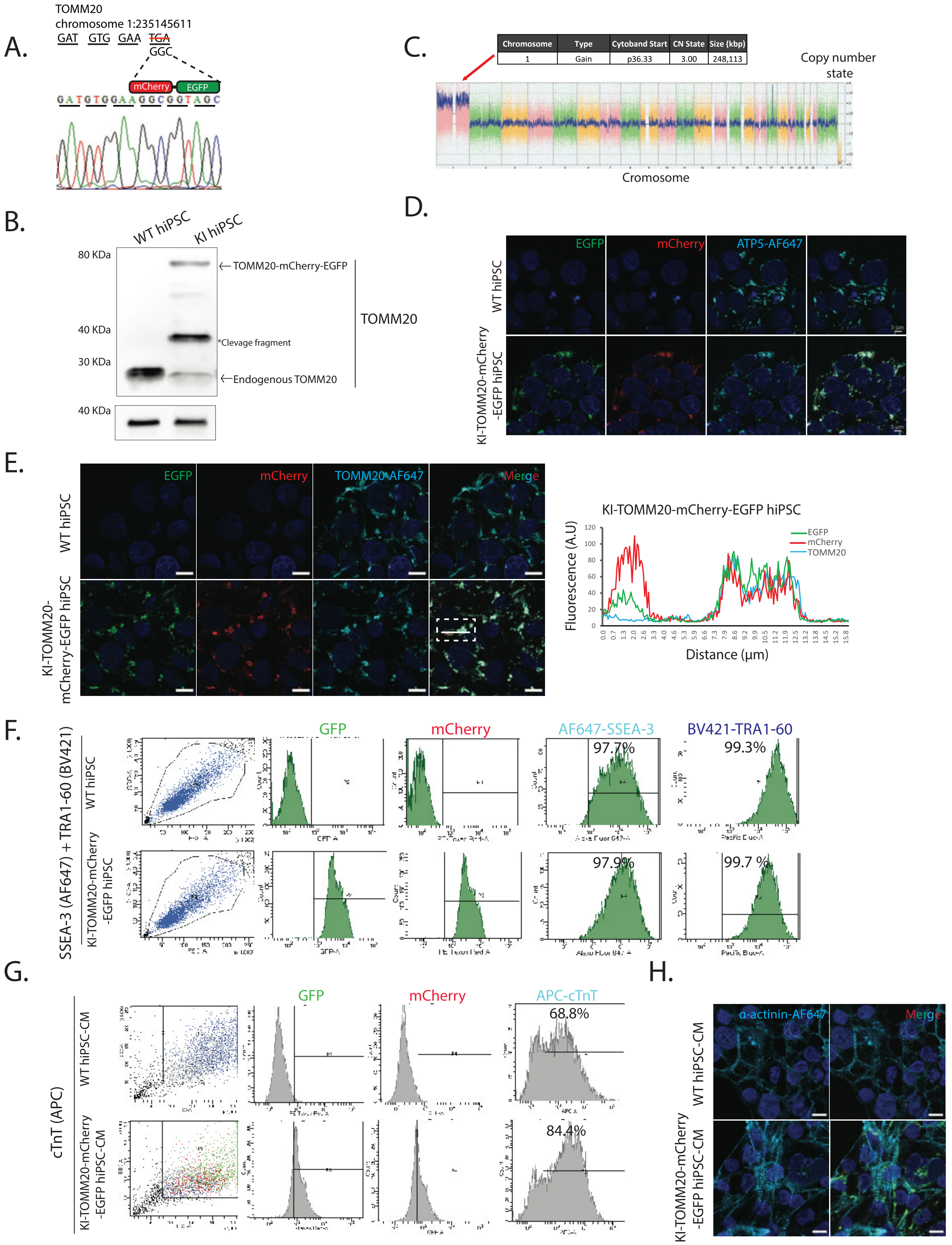

Journal of Cell Science • Supplementary information

Fig. S2. Characterization of the knock-in (KI)-TOMM20-mCherry-EGFP hiPSC line and derived cardiomyocytes.

A. Sanger sequencing of KI-TOMM20-mCherry-EGFP hiPSC line confirming the correct insertion of the tandem fluorescent tag (mCherry-EGFP) after the 3´end of the *Tomm20* gene after CRISPR/Cas9 genome editing. The TGA stop codon of *TOMM20* is indicated and was edited as shown.

B. Western blot analysis of TOMM20 protein expression levels in WT hiPSCs and KI-TOMM20-mCherry-EGFP hiPSCs. GADPH is used as a loading control. Representative blot of three independent experiments.

C. Karyotyping of KI-TOMM20-mCherry-EGFP hiPSCs was analysed using Kariostat+ analysis. Chromosomal aberration is indicated in the table. The whole genome view displays all somatic and sex chromosomes in one frame indicating their copy numbers. A value of 2 represents a normal copy number state (CN). A value of 3 represents chromosomal gain. The chromosome aberration is indicated by the red arrow for chromosome 1 (trisomy 1).

D. WT hiPSC and KI-TOMM20-mCherry-EGFP hiPSC were immunostained against ATP5 (cyan) and stained with DAPI (blue). The images are representative of two independent experiments. Scale bar: 5 µm.

E. WT hiPSC line and KI-TOMM20-mCherry-EGFP hiPSC reporter line were immunostained against TOMM20 (cyan) and stained with DAPI (blue). A line profile for mCherry and EGFP fluorescence as well as TOMM20 staining is indicated. The images are representative of two independent experiments. Scale bar: 10 µm

F. Flow cytometry analysis of the stemness markers (SEEA3 and TRA1-60) in WT hiPSC line and KI-TOMM20-mCherry-EGFP hiPSCs was conducted to confirm pluripotency after CRISPR/Cas9 genetic modification. Representative results of three independent experiments.

G. Flow cytometry analysis of the cTnT cardiac marker in WT hiPSC and KI-TOMM20-mCherry-EGFP hiPSC-derived cardiomyocytes was performed to confirm efficient cardiomyocyte differentiation in the genetically modified hiPSCs. Representative images of three independent experiments.

H. WT and KI-TOMM20-mCherry-EGFP hiPSC-derived cardiomyocytes (hiPSC-CMs) were immunostained against α-actinin (cyan) and stained with DAPI (blue). Representative confocal images of three independent experiments, with cardiomyocytes from a distinct hiPSC differentiation. Scale bar: 10 µm

Journal of Cell Science • Supplementary information

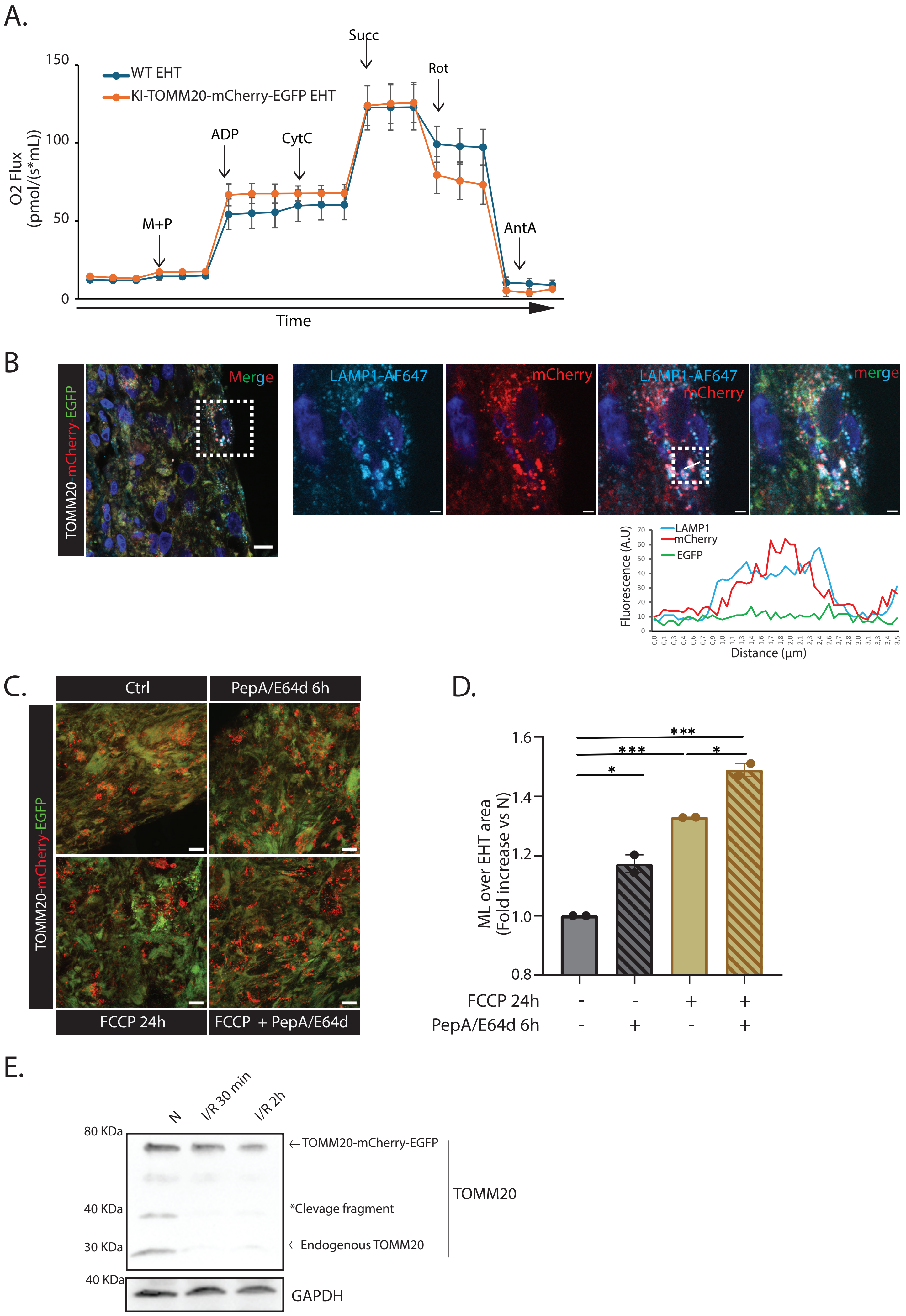

Journal of Cell Science • Supplementary information

Fig. S3. Characterization of KI-TOMM20-mCherry-EGFP EHTs.

A. Representative Oxygen consumption rates (OCR) in WT and KI-TOMM20-mCherry-EGFP EHTs. Addition of 0.1 mM malate (M) and 5mM pyruvate (P) indicates non-phosphorylating resting state (LEAK state). To test OXPHOS capacity 2.5 mM ADP was added. The integrity of the mitochondrial outer membrane was assessed by adding 10 µM cytochrome C (CytC); Incorporation of 100 mM succinate (Succ) demonstrates Complex I function; Addition of 0.5 µM rotenone (Rot) indicates complex II functions. Treatment with 2.5 µM antimycin (AntA) indicates residual oxygen consumption. The data represents three independent experiments.
B. TOMM20-mCherry-EGFP EHT sections were immunostained against LAMP1A (cyan) and stained with DAPI (blue). Insets show magnified views of the boxed area. A line profile for mCherry, and EGFP fluorescence as well as the LAMP1A staining is indicated. The images are representative of three independent experiments.
C. Representative confocal images of TOMM20-mCherry-EGFP EHTs treated with FCCP (10 µM) for 24 h, PepA (10 µg/ml) and E64d (10 µg/ml) for 6 h or for 24 h with FCCP with addition of Pep/E64d the last 6 hours of the FCCP treatment.
D. The number of red-only dots, corresponding to mitolysosomes (ML), was quantified and normalized to EHT area using mQC counter plugin for ImageJ. Results are expressed as a fold increase vs control (N). The data represents two independent experiments.

Data information: For each independent experiment the EHTs were generated with cardiomyocytes from a distinct hiPSC differentiation. Scale bar: 10 µm (B and C) 2 µm insets (B).

Journal of Cell Science • Supplementary information

A.

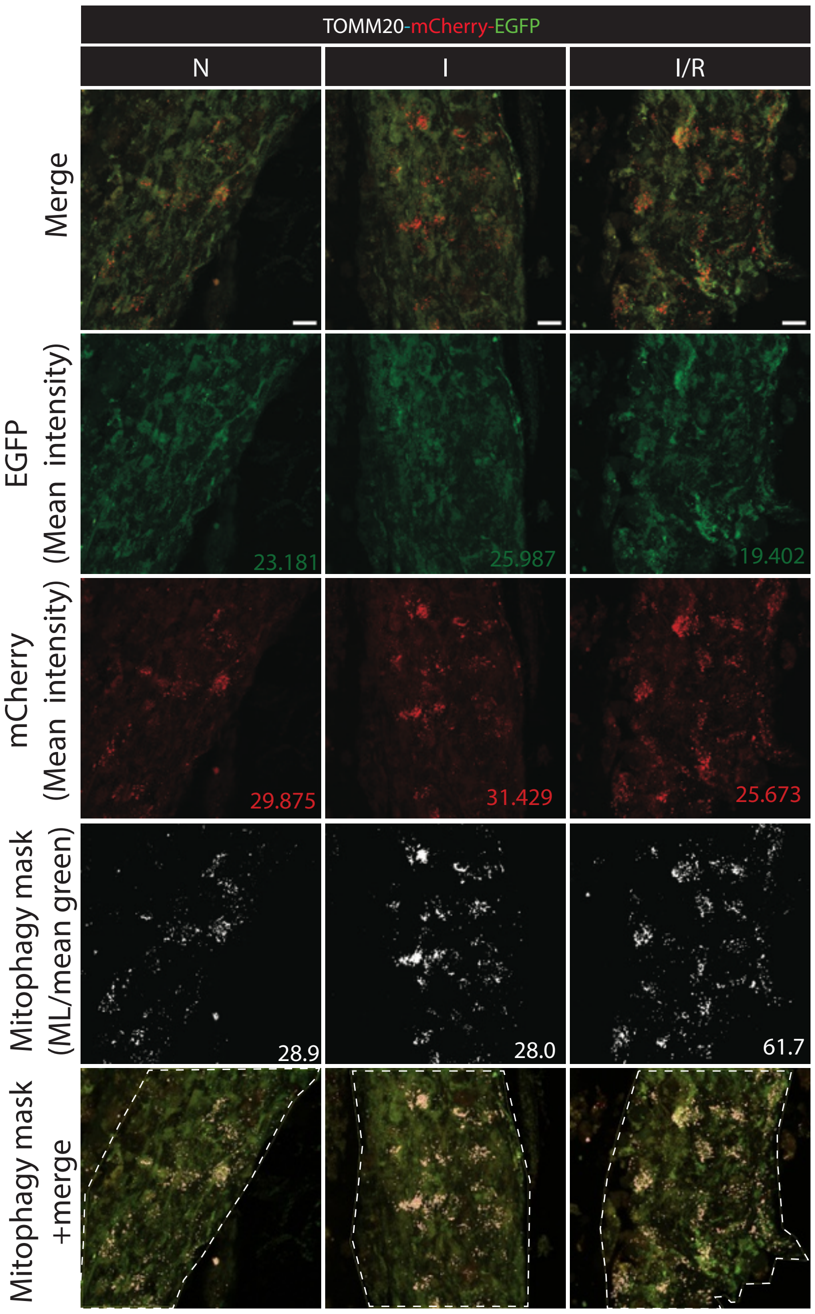
TOMM20-mCherry-EGFP
N
I
I/R
Merge
EGFP (Mean intensity)
23.181
25.987
19.402
mCherry (Mean intensity)
29.875
31.429
25.673
Mitophagy mask (ML/mean green)
28.9
28.0
61.7
Mitophagy mask +merge

B.

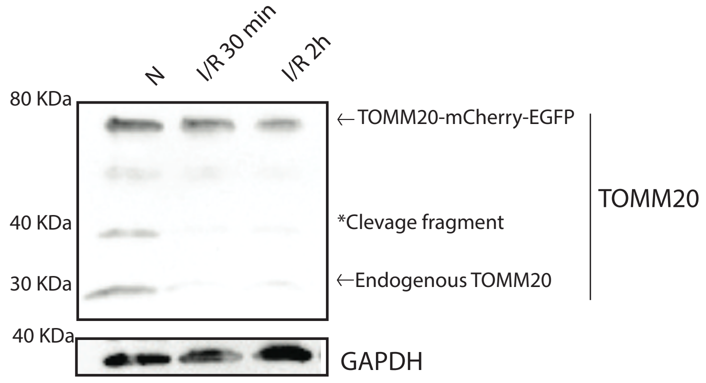
N
I/R 30 min
I/R 2h
80 KDa
40 KDa
30 KDa
40 KDa
←TOMM20-mCherry-EGFP
*Clevage fragment
←Endogenous TOMM20
TOMM20
GAPDH

Fig. S4. Mitophagy quantification and Western blot analysis of the mitophagy reporter (TOMM20-mCherry-EGFP) EHTs during I/R.

A. Confocal images corresponding to main Fig. 5C are displayed as merged images (top row), single mCherry and EGFP channels (second and third row). The measured fluorescence intensity in each channel for all the groups is displayed (numbers in lower right corners). The mitophagy mask (black and white) image created by the mito-QC Counter macro used for quantification is displayed (fourth row). The numbers displayed in the lower right corners reflect the total number of mitolysosomes (ML) detected by the macro divided by the mean green intensity in the ROI. The mitophagy mask is depicted as an overlay in the merged images where the ROI is indicated with white dashed lines (bottom row). Quantification was performed with the following parameters: Radius for smoothing images = 1; Ratio threshold = 1; Red channel threshold = Mean + 1 stdDev. Scale bar: 10 µm
B. Western blot using the anti-TOMM20 antibody to visualize the expression level of the TOMM20-mCherry-EGFP fusion protein and the endogenous TOMM20 protein in control conditions (N) and during I/R simulation with 30 min and 2 h of reperfusion. GAPDH is displayed as a loading control. The data represents two independent experiments.

Fig. 4E

ULK1

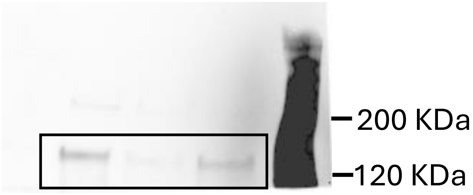

BNIP3

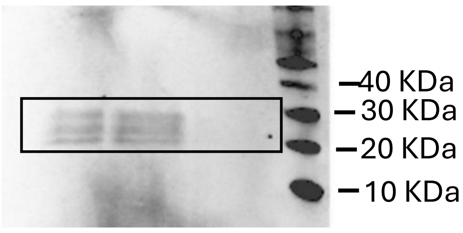

GAPDH (ULK1 and BNIP3 memb)

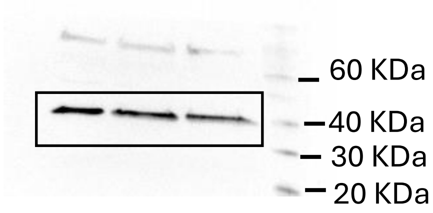

Journal of Cell Science • Supplementary information

PARKIN

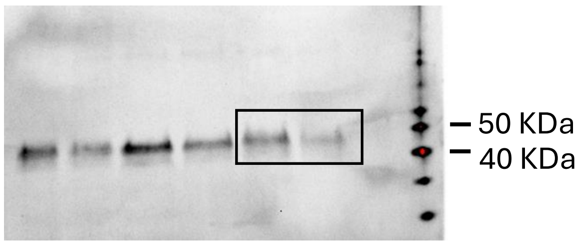

GAPDH (PARKIN memb)

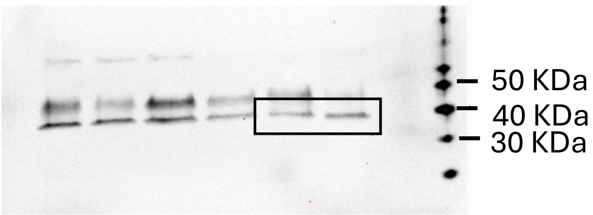

Fig S1

p62

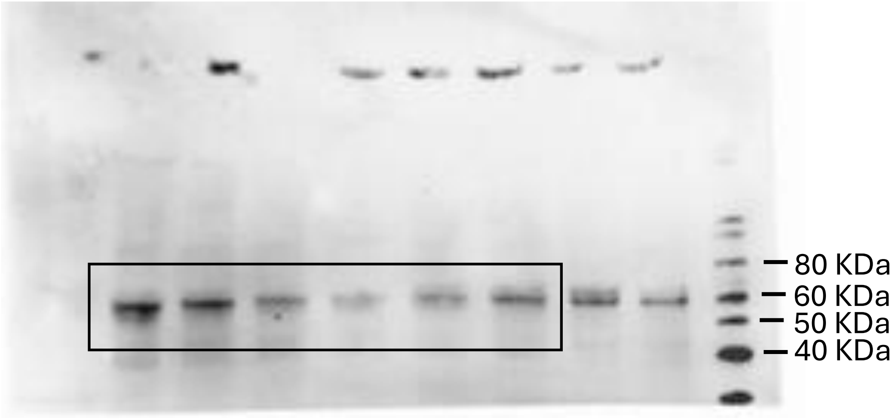

LC3B

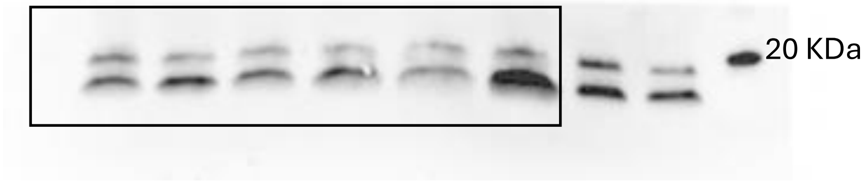

Journal of Cell Science • Supplementary information

GAPDH

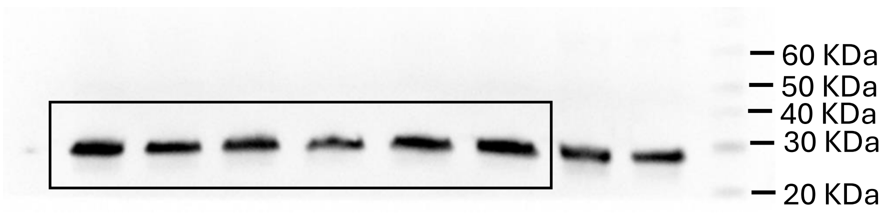

FIG S2B

TOMM20

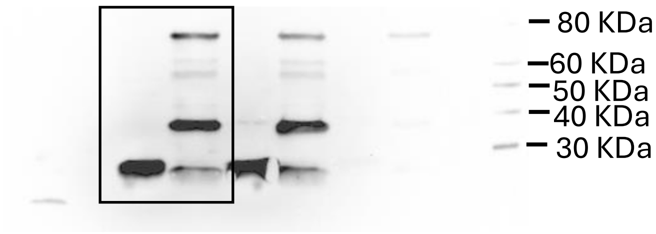

GAPDH

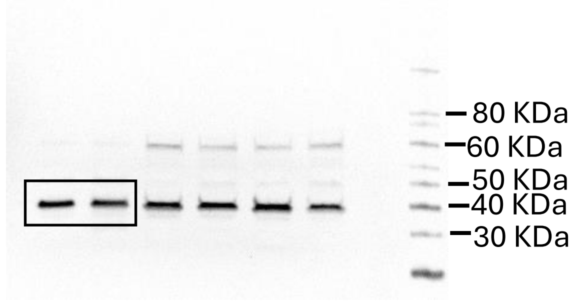

FIG S3E

TOMM20

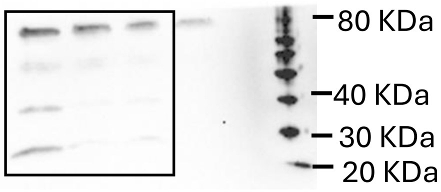

GAPDH

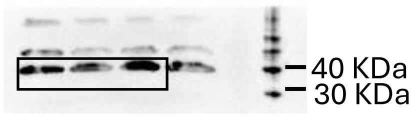

Fig. S5. Blot transparency

Table S1. List of autophagy modulators

Bafilomycin 1A	Sigma Aldrich	B1793
Pepstatin A	Sigma Aldrich	B5318
E64d	Sigma Aldrich	E8640
MRT68921 (ULK1 inhibitor)	Adooq Bioscience	A15880

Table S2. List of antibodies used for different purposes

**Antibodies**	**Reference or Source**	**Identifier or catalogue number**
**Immunofluorescence**		
α-actinin	Abcam	ab9465
cTnT	Abcam	ab91605
TOMM20	Santa Cruz	sc-11415
LAMP1	Abcam	ab25630
HIF1α	ThermoFisher	PA1-16627
ATP5	Abcam	ab14748
Goat anti-Rabbit IgG (H+L) Cross-Adsorbed Secondary Antibody, Alexa Fluor™ 488	Invitrogen	A11008
Goat anti-Mouse IgG (H+L) Cross-Adsorbed Secondary Antibody, Alexa Fluor™ 647	Invitrogen	A32728
Goat anti-Rabbit IgG (H+L) Cross-Adsorbed Secondary Antibody, Alexa Fluor™ 647	Invitrogen	A21244
**Western Blot**		
LC3B	Sigma Aldrich	L7543
p62 (SQSTM1)	BD Bioscience	610833
ULK1	Cell Signalling Technology	8054
PARKIN	Cell Signalling Technology	2132
BNIP3	Santa Cruz Biotechnologies	Sc-56167
TOMM20	Santa Cruz Biotechnologies<br>Abcam	sc-11415<br>ab186734
GADPH	Sigma Aldrich	G9545
HRP Goat Anti-Rabbit IgG	BD Biosciences	554021
HRP Goat Anti-Mouse IgG	BD Biosciences	554002
**Flow cytometry**		
SSEA-3 (AF647)	BD Biosciences	561145
TRA 1-60 (BV241)	BD Biosciences	562711
Isotype control AF647	BD Biosciences	560892
Isotype control BV241	BD Biosciences	562704
cTnT (APC)	Miltenyi Biotec	130-120-403
Isotype (REA) control APC	Miltenyi Biotec	130-113-446

Journal of Cell Science • Supplementary information
