# Supplementary material for: Mitophagy is induced in human engineered heart tissue after simulated ischemia and reperfusion
Source: J Cell Sci. 2025 Mar 19;138(9):jcs263408. doi: 10.1242/jcs.263408 (PMC11959618; doi:10.1242/jcs.263408)
Supplement: Supplementary file 1 [file joces-138-263408_review_history.pdf]

Journal of Cell Science

Mitophagy is induced in human engineered heart tissue after simulated ischemia and reperfusion

Mireia Nager, Kenneth B. Larsen, Zambarlal Bhujabal, Trine B. Kalstad, Judith Rössinger, Truls Myrmel, Florian Weinberger and Asa B. Birgisdottir
DOI: 10.1242/jcs.263408

Editor: Heidi McBride

Review timeline

Original submission:	26 June 2024
Editorial decision:	18 September 2024
First revision received:	11 December 2024
Editorial decision:	9 January 2025
Second revision received:	14 January 2024
Accepted:	30 January 2025

Original submission

First decision letter

MS ID#: jcs.263408

MS TITLE: Mitophagy is induced in human engineered heart tissue after simulated ischemia and reperfusion

AUTHORS: Mireia Nager; Kenneth B. Larsen; Zambarlal Bhujabal; Trine B. Kalstad; Judith Rössinger; Truls Myrmel; Florian Weinberger; Asa Birna Birgisdottir

We apologize for the long delay in review of your manuscript. After many reminders and attempts, we could not obtain a second review for your study. Reviewer 1 provided a thoughtful review and I have gone through the manuscript to finalize an editorial decision.

As you will see from their review, there are weaknesses in the study that would preclude publication in this form. I appreciate the importance of the development of this new gene edited TOM20 mitophagy reporter line to monitor mitochondrial protein turnover in human heart tissue, and the clinical importance of ischemia/reperfusion models. However, given that the primary focus of this study is the generation and validation of this tool, it is essential that the process of mitophagy be examined with both positive and negative controls. The kinetics and extent of mitochondrial turnover is not established here, and it was not confirmed whether the arrival of the probe in lysosomes was through mitophagy. The reviewer indicated that the study would be of interest if there were evidence (one way or another) that enhancement/prevention of mitophagy would alter the outcome of survival (or function) during ischemia/reperfusion. I agree with their general point, however in my opinion a deeper validation of the probe, and confirmation of mitophagy pathways active in this system would be an important contribution.

Reviewer 1

Advance summary and potential significance to field

The authors employed human engineered heart tissue to assess the mitochondrial dysfunction and mitophagy during ischemia and reperfusion in human hearts. The study also employed the a

© 2025. Published by The Company of Biologists under the terms of the Creative Commons Attribution License (https://creativecommons.org/licenses/by/4.0/).

mitochondria targeted fluorescent protein marker with mCherry and GFP that can assess mitophagy. The novel methodology may enable novel therapeutic strategies and allow drug testing in these engineered heart tissues.

Comments for the author

General:
Since the authors report the development of the new method, it would be important to include positive and negative controls in their assays. It would be helpful if the authors showed the effect of autophagy suppression, such as Atg7 or Parkin downregulation (negative controls) and mitophagy stimulation (positive controls, CCCP) in their assay system. It would also be important for the authors to show several time points since the time course of mitophagy after reperfusion could be complex.

Please provide quantification of the staining and EM data. It would be important to show that the authors' statement is not based upon one experiment. Many are presented without quantification.

In Figure 3B, what is the mechanism by which I/R + BafA1 markedly upregulates LAMP1? This alone may not suggest that "mitophagy is induced during reperfusion" (page 6, line indicators 55-56). In their PLA experiments, the authors should show the staining with anti-LAMP1 alone and anti-TOMM20 alone.
What is the overall stability of TOMM20-mChery-EGFP in response to ischemia reperfusion. Does it undergo mitophagic degradation? If so, what is the kinetics of degradation?

The authors could have addressed the role of mitophagy in testing the effect of an intervention to modulate the level of mitophagy in the context of reperfusion, which would add some novelty to this study. This would be the best proof that the method can be used to prove the function of mitophagy in their model.

Specific comments:
1) The Figure 2B and C, shows defects in mitochondrial respiration and ATP production but it would be important to understand the level of uncoupled respiration in the tissues with FCCP or any uncouplers to reveal the maximal respiration capacity.
2) The trisomy of chromosome 1 due to the mitophagy reporter insertion discussed in the study has very few effects and proves to be a good model for studying mitophagy. However, it may need to be verified for a variety of cellular responses, including tumorigenicity and altered metabolic activity.

First revision

Author response to reviewers' comments

Response from the authors to the editor and the reviewer.

Comments from the Guest editor (Heidi McBride):

I appreciate the importance of the development of this new gene edited TOM20 mitophagy reporter line to monitor mitochondrial protein turnover in human heart tissue, and the clinical importance of ischemia/reperfusion models. However, given that the primary focus of this study is the generation and validation of this tool, it is essential that the process of mitophagy be examined with both positive and negative controls. The kinetics and extent of mitochondrial turnover is not established here, and it was not confirmed whether the arrival of the probe in lysosomes was through mitophagy. The reviewer indicated that the study would be of interest if there were evidence (one way or another) that enhancement/prevention of mitophagy would alter the outcome of survival (or function) during ischemia/reperfusion. I agree with their general point, however in my opinion a deeper validation of the probe, and confirmation of mitophagy pathways active in this system would be an important contribution.

© 2025. Published by The Company of Biologists under the terms of the Creative Commons Attribution License (https://creativecommons.org/licenses/by/4.0/).

Author reply:

We are very pleased that the editor appreciates the importance of developing the hiPSC TOMM20 mitophagy reporter line to monitor mitochondrial protein turnover in human engineered heart tissue (EHT) and the clinical relevance of ischemia/reperfusion models.

We agree with the editor that we should have included controls to validate that we are monitoring mitophagy using the tandem-tagged TOMM20 protein. Thus, as explained in the response to the reviewer (see below) we have now included siRNA knockdowns of established mitophagy regulators (ULK1, BNIP3 and Parkin) using TOMM20-mCherry-EGFP hiPSC derived cardiomyocytes (Fig. 4E, F). Moreover, we have included a FCCP control in the EHTs (Fig.S3C, D) and used an ULK1 inhibitor to assess the effect during I/R in the EHTs (Fig. 5G, H). We believe that these new data support that our pH-sensitive probe allows for mitophagy monitoring and that parallel mitophagy processes with functional redundancy are responsible for the arrival of the probe to lysosomes in the hiPSC-derived cardiomyocytes during normal culture conditions. However, as with other pH-sensitive mitophagy reporters such as mitoQC and mtKeima, we cannot exclude the involvement of other forms of mitochondrial engulfment into lysosomes. We have now stated this in the discussion part on page 11. Our results with the ULK1 inhibitor during I/R demonstrate the involvement of the core autophagy machinery in induced mitophagy during reperfusion. We hope that the editor finds this sufficient as a deeper validation of the probe and mitophagy pathways.

To address the kinetics and the extent of mitochondrial turnover, we performed a time-course experiment in the EHTs with 20, 40, and 60 min of reperfusion in addition to the original 2 h timepoint. To assess mitophagy flux, we also included the lysosomal inhibitors Pepstatin A (PepA) and E64d during the 2 h reperfusion timepoint (Fig. 5E, F). PepA and E64d need relatively long time to display an effect and thus were not included in the shorter reperfusion timepoints. Notably, we see a rapid increase in the number of red-only dots after only 20 min of reperfusion, while there is a gradual slight reduction until 2 h (but still significantly higher than the normoxia control). Interestingly, the presence of PepA/E64d during 2 h of reperfusion indicates that the flux of lysosomal degradation of mitochondria indeed is induced, reflected by an increase in the abundance of mitolysosomes, above the detected level with PepA/E64d in the normoxia time-matched control. Furthermore, western blots of the TOMM20- mCherry-EGFP fusion protein display a reduction in the protein level after I/R simulation in EHTs, indicative of degradation (Fig. S3E).

Delineating the active mitophagy processes involved during ischemia and reperfusion in the EHTs would require the construction of several knockouts in the KI-TOMM20-mCherry-EGFP hiPCS line, given the siRNA knockdown results, and we feel is beyond the scope of this paper. The effect of the ULK1 inhibitor (Fig. 5G, H) shows that the activity ULK1, one of the most upstream effectors of different mitophagy pathways, is important. Furthermore, determining whether induced mitophagy during reperfusion is cardioprotective or not is difficult to assess. Our data represent an important starting point for future studies to decipher if further inducing or inhibiting mitophagy during reperfusion is beneficial in a therapeutic setting.

Comments from the Reviewer:

Reviewer 1: The authors employed human engineered heart tissue to assess the mitochondrial dysfunction and mitophagy during ischemia and reperfusion in human hearts. The study also employed a mitochondria targeted fluorescent protein marker with mCherry and GFP that can assess mitophagy. The novel methodology may enable novel therapeutic strategies and allow drug testing in these engineered heart tissues.

General:

Since the authors report the development of the new method, it would be important to include positive and negative controls in their assays. It would be helpful if the authors showed the effect of autophagy suppression, such as Atg7 or Parkin downregulation (negative controls) and mitophagy stimulation (positive controls, CCCP) in their assay system. It would also be important for the authors to show several time points since the time course of mitophagy after reperfusion

© 2025. Published by The Company of Biologists under the terms of the Creative Commons Attribution License (https://creativecommons.org/licenses/by/4.0/).

could be complex.

Author response:

We thank the reviewer for pointing out the importance of demonstrating positive and negative controls. In Fig. 5B, C (now Fig. 5A, B), we already did show the opposite effects of the lysosomal inhibitors Pepstatin A (PepA)/E64d (increase) and Bafilomycin A1 (BafA1) (decrease) for the presence of red- only dots in EHTs, indicating the prerequisite of functional lysosomes for the appearance of red-only structures. We have now included an experiment using FCCP treatment of EHTs as a positive control of mitophagy induction. Furthermore, we also included PepA/E64d in the last hours of the FCCP treatment to verify the induction of mitophagy flux with FCCP. These data are presented in Fig. S3C, D.

For the suggested negative controls, such as downregulation of Atg7 or Parkin, we applied siRNA knockdowns in the hiPSC-derived TOMM20-mCherry-EGFP cardiomyocytes in 2D cultures. In Fig. 4F we demonstrate that siRNA knockdown (72 hours) of ULK1, BNIP3 or Parkin in the cardiomyocytes leads to a significant decrease in the number of acidified (red-only) mitochondria. Furthermore, the inclusion of PepA/E64d for the last 2 hours of the knockdowns indicates that the mitophagy flux is indeed inhibited since there is no increase of red-only dots in the presence of the siRNAs. These results demonstrate the importance of the core-autophagy machinery (ULK1) as well as the mitophagy receptor BNIP3 and the known mitophagy mediator Parkin, in the formation of acidic mitochondria in the cardiomyocytes. Thus, the mitophagy process, visualized through the pH-sensitive tandem tagged TOMM20, appears to be governed by several parallel mechanisms.

To address the time course of mitophagy during reperfusion of TOMM20-mCherry-EGFP EHTs we conducted reperfusion for shorter timepoints, including 20, 40 and 60 min in addition to the previously used 2 h reperfusion timepoint. Furthermore, to assess mitophagy flux we also included the lysosomal inhibitors PepA/E64d during the 2 h reperfusion timepoint. The results indicate a rapid significant increase in red-only dots (lysosomal degradation of mitochondria) after only 20 min of reperfusion. Intriguingly, in the presence of PepA/E64d during 2 h of reperfusion the abundance of acidic mitochondria has increased even further, indicative of induced flux. These results are displayed in Fig. 5E, F.

Please provide quantification of the staining and EM data. It would be important to show that the authors' statement is not based upon one experiment. Many are presented without quantification.

Author response:

We thank the reviewer for pointing out the importance of displaying quantification of immunostaining (when applicable) and to include information regarding the number of experiments conducted.

We have now included a quantification of the HIF1a immunostaining in Fig. 1C and provided line profiles for the cTnT staining in Fig. 1F to emphasize the detected disarray of sarcomeres during ischemia. For confocal images demonstrating co-localization of the EGFP/mCherry fluorophores and TOMM20 staining, we have included line profiles (Figs. 4C and S2E). In the figure legends we have now stated the number of different experiments conducted where this information was previously lacking.

Regarding the EM data, the TEM images (Fig. 2A and Fig. 3E) are from representative experiments and were not acquired for quantification purposes. The TEM images are displayed to indicate qualitative observations. Thus, we have modified the text accompanying Fig. 3E on page 7 and removed the phrase “higher abundance of lysosomes” as that would indicate quantification of the number of lysosomes. Instead, we use the phrase “indicate more pronounced proximity”.

In Figure 3B, what is the mechanism by which I/R + BafA1 markedly upregulates LAMP1? This alone may not suggest that "mitophagy is induced during reperfusion" (page 6, line indicators 55-56). In their PLA experiments, the authors should show the staining with anti-LAMP1 alone and anti-

© 2025. Published by The Company of Biologists under the terms of the Creative Commons Attribution License (https://creativecommons.org/licenses/by/4.0/).

TOMM20 alone.

Author response:

Since fusion of autophagosomes and lysosomes is inhibited by BafA1, as well as degradation of lysosomal contents, accumulation of lysosomes and in particular expansion of the degradative compartment would be expected (see e.g. Mauthe et al., 2018; doi: 10.1080/15548627.2018.1474314). This would be reflected in an increased signal for immunostaining of LAMP1. The increase of LAMP1 staining after I/R in the presence of BafA1 during the reperfusion is striking. We agree with the reviewer that LAMP1 staining alone does not indicate induced mitophagy. Therefore, we have included a western blot of the autophagy markers LC3 and p62 after I/R simulation of the EHTs with and without BafA1. The western blot is displayed in Fig. S1E. Since these data together with LAMP1 staining only infer autophagy flux induction during I/R we have removed the word “mitophagy” from the last sentence of the paragraph on page 7 and state that “autophagy flux is induced after I/R simulation”.

In our PLA experiment in Fig. 3C we have now included the negative control with the anti-LAMP1 antibody alone.

What is the overall stability of TOMM20-mCherry-EGFP in response to ischemia reperfusion. Does it undergo mitophagic degradation? If so, what is the kinetics of degradation?

Author response:

To investigate the overall stability of the TOMM20-mCherry-EGFP fusion protein during I/R we performed western blots of EHT total protein lysates from different reperfusion times (30 min and 2 hours) and compared with lysates from control EHTs. The anti-TOMM20 blot both demonstrates a reduction in the fusion protein level after 30 min as well as after 2 hours of reperfusion. These data are presented in Fig. S3E and indicate that the pH-sensitive probe is being degraded during reperfusion. In addition, as mentioned above, the time-course experiments in EHTs subjected to 20, 40, 60 min or 2 h of reperfusion after ischemia (Fig. 5E, F) indicates induced mitophagy already after 20 min.

The authors could have addressed the role of mitophagy in testing the effect of an intervention to modulate the level of mitophagy in the context of reperfusion, which would add some novelty to this study. This would be the best proof that the method can be used to prove the function of mitophagy in their model.

Author response:

The role of mitophagy during reperfusion, i.e. whether mitophagy is cardioprotective or not, is still an unanswered question in cardiovascular research and we feel is beyond the scope of this manuscript. To investigate the involvement of ULK1 (a core autophagy protein) in induced mitophagy during I/R simulation in the TOMM20-mCherry-EGFP EHTs, we conducted experiments with pretreatment of the EHTs with a ULK1 inhibitor before being subjected to I/R simulation. This is presented in Fig. 5G, H. Our results indicate that ULK1 activity is needed for mitophagy induction during I/R. However, considering the siRNA knockdown experiments (Fig. 4F) we suspect the involvement of several parallel mitophagy mechanisms with functional redundancy at play also during I/R. Thus, future studies will be needed to address this.

Specific comments:

1) The Figure 2B and C, shows defects in mitochondrial respiration and ATP production but it would be important to understand the level of uncoupled respiration in the tissues with FCCP or any uncouplers to reveal the maximal respiration capacity.

Author response:

© 2025. Published by The Company of Biologists under the terms of the Creative Commons Attribution License (https://creativecommons.org/licenses/by/4.0/).

We have now included the use of FCCP during the respiration experiment to reveal the maximal respiration capacity. The results are presented in Fig. 2D and demonstrate a significant reduction in maximal respiration after I/R.

2) The trisomy of chromosome 1 due to the mitophagy reporter insertion discussed in the study has very few effects and proves to be a good model for studying mitophagy. However, it may need to be verified for a variety of cellular responses, including tumorigenicity and altered metabolic activity.

Author response:

We agree with the reviewer that tumorigenicity of trisomy 1 could be an issue for *in vivo* studies. However, we did not investigate tumorigenicity of trisomy 1 in our hiPSCs as this is not our area of expertise, and in our opinion beyond the scope of this manuscript. Regarding metabolism, Madsen et al. 2020 (doi: https://doi.org/10.1161/CIRCULATIONAHA.119.04444) detected trisomy 1 in the same hiPSC line as we used. The authors measured glucose consumption and lactate production in the derived EHTs and did not find differences from the WT. We also conducted mitochondrial respiration analysis in TOMM20-mCherry-EGFP EHTs and compared with the WT EHTs. These data are now included in Fig.S3A and we did not detect any significant differences between the WT and the genetically modified EHTs.

Second decision letter

MS ID#: jcs.263408

MS TITLE:Mitophagy is induced in human engineered heart tissue after simulated ischemia and reperfusion

AUTHORS:Mireia Nager; Kenneth B Larsen; Zambarlal Bhujabal; Trine B Kalstad; Judith Rössinger; Truls Myrmel; Florian Weinberger; Asa Birna Birgisdottir
ARTICLE TYPE:Research Article

Thank you for the detailed resubmission of your manuscript. The reviewer is very favourable now, but still had a few important points that should be clarified before we can accept it. The few points are to clarify how the imaging data was quantified and normalized, as you will see from their detailed review. I hope that you will be able to address their points because we believe the study will be of interest to the JCS readership in our special issue.

Reviewer 1

Advance summary and potential significance to field

Establishment of the method to quantify mitophagy in human engineered heart tissue

Comments for the author

The authors are very responsive and the paper is improved.
Despite their effort, some raw data are not very impressive to me. Some confocal images may not be as representative as they should be and may be misleading. One example is Fig. 5C, the change in the "I/R" sample may not be very impressive compared to the control (N) sample. Please clarify the method of quantification. What is the difference between "ML over mitochondria content" and "ML area over mitochondrial content" in Figure 5? Why are the values of ML over mitochondria content of PepA/E64d 2h samples in Fig. 4F (around 1.3) and 5B (close to 2) different? In Fig. 5C, it would be helpful if the authors showed the overall level of green, red, and green+red. The change

© 2025. Published by The Company of Biologists under the terms of the Creative Commons Attribution License (https://creativecommons.org/licenses/by/4.0/).

in the "I/R" sample may not be so clear compared to control (N). It is unclear why both red and green are increased in the "I" sample. Is this because the sample just expresses more reporters? If the level of the reporter differs from sample to sample, does it affect the quality of the assay?

Second revision

Author response to reviewers' comments

Reviewer 1:

The authors are very responsive and the paper is improved.

Autohor reply:
We are very pleased that the reviewer appreciates our efforts and thinks that our paper is improved.

Despite their effort, some raw data are not very impressive to me. Some confocal images may not be as representative as they should be and may be misleading. One example is **Fig. 5C**, the change in the "I/R" sample may not be very impressive compared to the control (N) sample.

Author reply:
We thank the reviewer for pointing this out. The images in Fig.5C were chosen for display as representative images for each condition because the quantification values of mitolysosomes in these images are around the measured mean values for each group. We agree with the reviewer that the image chosen for the I/R group does not present a dramatic difference from the normoxia (N) group and thus we have changed the representative image for the I/R group in Fig. 5C to better visually reflect the quantified almost 2-fold increase in mitolysosomes. It should be noted that the quantification reflects results from 5 independent experiments.

Please clarify the method of quantification. What is the difference between "ML over mitochondria content" and "ML area over mitochondrial content" in Figure 5?

Author reply:
As stated in the Materials and methods section on page 18 and in the Figure legend to Figure 5 on page 31 and 32, we used the published mito-QC Counter macro for FIJI/ImageJ (Montava-Garriga et al. 2019doi: 10.1016/j.mad.2019.111196) for quantification of red-only dots/mitolysosomes (ML). Here, it is the ratio of red fluorescence over green fluorescence that influences the ML count which then is normalized against total mitochondrial content per image. The first parameter “ML over mitochondrial content” (“nML_over_green” in the macro measurements) reflects the number of detected red-only dots/mitolysosomes (ML) divided by green mean intensity (mitochondrial content) per image in each treatment group (N, I and I/R). “ML area over mitochondrial content” (“MLarea_µm2_over_green” in the macro measurements) reflects the total measured area of all the ML/red only dots divided by green mean intensity (mitochondrial content) per image. The reason to show the total ML area is that mitolysosomes can increase in size when mitophagy is induced, which may not be directly reflected by the total ML number. This also helps to safeguard against segmentation artifacts when detecting separate red-only dots. The values presented in the graphs in Fig. 5D are averages from at least 8 images (each ROI corresponds to an average of 7000 μm^2 area) per group in 8 independent experiments (total of at least 64 images per group were analysed) using engineered heart tissue (EHT) with cardiomyocytes from distinct hiPSC differentiations. To render the quantification more accessible, we have added more explanation of the macro quantification to the Materials and methods section on page 18 and adjusted the legend to Fig. 5D.

Why are the values of ML over mitochondria content of PepA/E64d 2h samples in Fig. 4F (around

© 2025. Published by The Company of Biologists under the terms of the Creative Commons Attribution License (https://creativecommons.org/licenses/by/4.0/).

1.3) and 5B (close to 2) different?

Author reply:
The reason for this difference in values is that the samples analyzed in Fig. 4F are cardiomyocytes in 2D culture while the samples analyzed in Fig. 5B are vibratome sections of EHTs. Thus, the effect of 2 hours treatment with PepA/E64d is more pronounced in cardiomyocytes in 3D culture (EHT) vs 2D cardiomyocytes. Possibly, this is due to that cardiomyocytes in 3D (EHT) performing work rely more on OXPHOS than cardiomyocytes in 2D culture (Ulmer et al. 2018; doi: 10.1016/j.stemcr.2018.01.039) and thus would accumulate more damaged mitochondria. We realized that the description of the quantification of ML in the 2D cardiomyocytes using the mQC Counter plugin for ImageJ was missing in the Materials and methods section on page 18 and in the legend to Figure 4F. We apologize for that and have now added this information in the revised version of the manuscript. The quantification approach is the same as for the EHTs but the area for normalization per image was the whole image frame for the cells and not a manually selected area as for the EHT sections.

In Fig. 5C, it would be helpful if the authors showed the overall level of green, red, and green+red. The change in the "I/R" sample may not be so clear compared to control (N).

Author reply:
To address this, we have added a new supplementary figure (Fig. S4B) where we show the single channels (EGFP and mCherry, respectively) of the merged images in Fig. 5C, indicating the measured fluorescence intensity in each channel for all the groups (numbers in lower right corners). In addition, we also display the mitophagy mask created in the macro. The mask reflects the total area and mean size of mitolysosomes as well as enabling visualisation of the quantified mitophagy areas. The numbers displayed in the lower right corners reflect the total number of mitolysosomes detected by the macro divided by the mean green intensity in the selected area. Furthermore, we show the mitophagy mask as an overlay on the merged images where the area selected for quantification is indicated with white dashed lines.

Please note that previous Fig.S3E has been moved and is now Fig. S4B to fit the presentation of the results in the main text of the manuscript.

It is unclear why both red and green are increased in the "I" sample. Is this because the sample just expresses more reporters? If the level of the reporter differs from sample to sample, does it affect the quality of the assay?

Author reply:
The density of cells per area is a bit different from area to area within each EHT, independent of treatment. The images are from vibratome sections of the EHTs and we imaged at least 8 positions per EHT section/sample in each experiment (total of 8 experiments) to ensure representation of different cell density areas. For each experiment, the EHTs subjected to the different treatments (N, I or I/R) are cast at the same time with the same cardiomyocytes and allowed to mature for at least 21 days in culture on beforehand. The visual increase in red and green fluorescence in the “I” sample in Fig. 5C is due to a more cell dense area in the EHT section displayed. Thus the “green mean” measured value representing total mitochondrial content will be higher in this image. Importantly, as mentioned above, it is the ratio of red fluorescence over green fluorescence that influences the ML count which then is normalized against total mitochondrial content per image. For better visual comparison, we have changed the representative image for the I group (in addition to the image for the I/R group) in Fig. 5C. Overall, the level of the reporter fluorescence intensity is quite consistent in the different batches of EHTs used in the different experiments. In general, the fluorescence intensity of the reporter is quite low since the reporter is endogenously encoded.

Third decision letter

© 2025. Published by The Company of Biologists under the terms of the Creative Commons Attribution License (https://creativecommons.org/licenses/by/4.0/).

MS ID#: jcs.263408

MS TITLE: Mitophagy is induced in human engineered heart tissue after simulated ischemia and reperfusion

AUTHORS: Mireia Nager; Kenneth B. Larsen; Zambarlal Bhujabal; Trine B. Kalstad; Judith Rössinger; Truls Myrmel; Florian Weinberger; Asa Birna Birgisdottir
ARTICLE TYPE: Research Article

I am happy to tell you that your manuscript has been accepted for publication in Journal of Cell Science, pending standard publication integrity checks.

© 2025. Published by The Company of Biologists under the terms of the Creative Commons Attribution License (https://creativecommons.org/licenses/by/4.0/).
